# Improved Computational Target Site Prediction for Pentatricopeptide Repeat RNA Editing Factors

**DOI:** 10.1371/journal.pone.0065343

**Published:** 2013-06-06

**Authors:** Mizuki Takenaka, Anja Zehrmann, Axel Brennicke, Knut Graichen

**Affiliations:** 1 Molekulare Botanik, Universität Ulm, Ulm, Germany; 2 Institut für Mess-, Regel- und Mikrotechnik, Universität Ulm, Ulm, Germany; NIGMS, NIH, United States of America

## Abstract

Pentatricopeptide repeat (PPR) proteins with an E domain have been identified as specific factors for C to U RNA editing in plant organelles. These PPR proteins bind to a unique sequence motif 5′ of their target editing sites. Recently, involvement of a combinatorial amino acid code in the P (normal length) and S type (short) PPR domains in sequence specific RNA binding was reported. PPR proteins involved in RNA editing, however, contain not only P and S motifs but also their long variants L (long) and L2 (long2) and the S2 (short2) motifs. We now find that inclusion of these motifs improves the prediction of RNA editing target sites. Previously overlooked RNA editing target sites are suggested from the PPR motif structures of known E-class PPR proteins and are experimentally verified. RNA editing target sites are assigned for the novel PPR protein MEF32 (mitochondrial editing factor 32) and are confirmed in the cDNA.

## Introduction

The in plants vastly expanded family of pentatricopeptide repeat (PPR) proteins provides diverse RNA maturation functions mostly to the two organelles mitochondria and plastids [1], [2]. RNAs synthesized in these organelles from their resident genomes are processed by intron excision, 5′- and 3′-terminal processing, endonucleolytic fragmentation and RNA editing. Different classes of PPR proteins are involved in all of these steps. About 200 of the 450 PPR proteins in flowering plants belong to a subfamily characterized by related C-terminal extensions (E-domains; [3], [4]). All PPR proteins identified to be involved in RNA editing belong to this class [5]. So far only one exception has been documented where a protein with only PPR repeats but no extension influences editing at several sites [6]. A number of PPR RNA editing proteins have been identified through analysis of mutants with phenotypic defects in organellar functions. Other analogous mutants do not show physiological phenotypes and require a direct comprehensive analysis of all editing sites. Since this is very labour and cost intensive particularly for the more than 400 sites in mitochondria, a tool to predict target sites from the sequence of a given candidate PPR protein will be very useful.

Previously, the strong sequence specific interactions between the non-extended PPR proteins and their target RNA have been used to determine features in the PPR repeats which correlate with the contacted nucleotide identities in the RNA [7]. Several of these non-extended PPR proteins have specific functions in internal and exonucleolytic RNA processing through tightly binding to the RNA at specific sites and to protect the thus covered termini and/or guide RNA processing enzymes.

In initial correlations, the three amino acid positions 1′, 4 and 6 (also labelled as ii, 1 and 4 in [8]) in the repeat elements of these PPR proteins were found to be occupied by amino acids whose identity shows some accord with the nucleotide moiety opposite the respective repeat unit [8], [9]. Amino acid position 1′ (or ii) is the first amino acid of the C-terminally adjacent repeat. These correlations were recently further refined [7], [9] and experimental evidence confirmed the conclusions [10]. In experimental assays, amino acids at these positions were altered in several PPR repeats and the manipulated PPR protein was found to indeed attach selectively to the predicted novel RNA sequence motif [10].

These analyses covered two types of the PPR motifs, the ‘regular’ ones with 35 amino acids (called P type [4]) and some of the shorter ones with 31–32 amino acids (S type). The longer repeats (L type) with 35–40 amino acids were not included. These L repeats may be important in the PPR proteins involved in RNA editing, since this subgroup uniquely consists of alternating P-L-S elements. In large PPR proteins with more than ten repeat elements not all of them may actually contact the RNA, one or more may function as spacers to allow for the 3D alignment of RNA and PPR repeats. Such gaps could compensate for different spatial lengths of the nucleotide chain and the PPR repeats in the proteins [8]–[10]. However, presently repeats looped out cannot be distinguished from those contacting the RNA. Furthermore, the PPR elements attaching to RNA and those not binding to a nucleotide may vary between different target sites of a given PPR protein [11]. In this contribution we include the L motifs in the alignment and find that this improves the prediction of the RNA target sites for a given PPR protein. Experimental analysis of respective mutants confirms the accuracy of the prediction for several known PPR proteins and allows assignment of the RNA editing target sites for the novel factor MEF32 (mitochondrial editing factor). We believe that this refined code improves the potential to generate specific RNA binding proteins for any sequence, analogous to the possibilities the TAL proteins offer to DNA manipulation [12].

## Methods

### Computational Analysis of RNA Editing Factors

**Figure 1:**
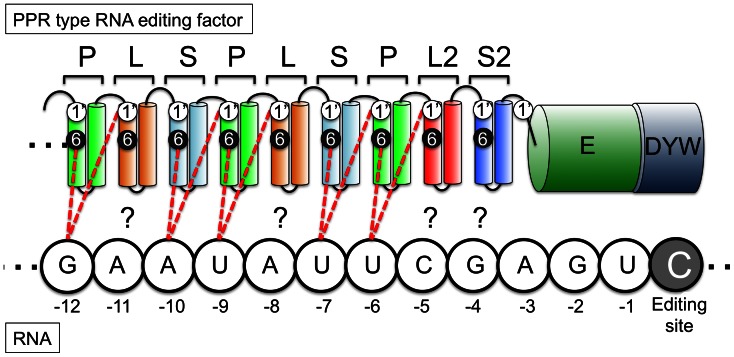
Structure model of RNA editing PPR proteins and their alignment to the RNA editing target sequence. The RNA editing PPR proteins are extended at their C-termini by E and often also by DYW domains. Different from P-type PPR proteins, the RNA editing PPR proteins contain alternating P-L-S type elements. The positions of the amino acid identities at positions 6 and 1′ are not given in the structurally correct position. These two amino acid positions have here been correlated to nucleotide identities (Figure S1). Dashed lines indicate their presumed connection to target nucleotide identities. Position 1′ is the first amino acid of the respective C-terminally adjacent repeat. For element S2 this position corresponds to amino acid 33 of this repeat while the E domain begins by convention only after amino acid 36. To illustrate this unclear assignment we placed position 1′ for the S2 element between the S2 and E domains. Question marks indicate the connections to the L, S2 and L2 domains investigated here for correlations with the opposite nucleotides. The nucleotide sequence is arbitrary and is spelled out solely to indicate the specific order of nucleotides here.

The L, L2 and S2 type motifs characteristic for RNA editing PPR proteins were not included in previous analyses. To investigate their potential contact with selected nucleotide identities, we analysed amino acid positions 6 and 1′ in all classes of repeat units in 41 PPR RNA editing factors and aligned them with the respective nucleotides in the upstream sequences of their target RNA editing sites (Figure 1 and Figure S1). Amino acids 6 and 1′ correspond to the sixth amino acid of the considered PPR motif and the first amino acid of the next C-terminal PPR motif which is accordingly termed 1′ (or 33), respectively (Figure 1 and Figure S1). To position the RNA, the fourth nucleotide upstream of each editing site (nucleotide –4) was aligned to the S2 motif. The S2 element is located directly N-terminal of the E motif. The PPR elements N-terminally following the S2 motif were aligned consecutively with the subsequent upstream (5′) nucleotides. In three separate considerations, amino acids at either position 6 or 1′ or the combination of amino acids at both positions were recorded with respect to the corresponding nucleotides.

For the computational analysis, a combinatorial search was performed by considering all possible alignments of the PPR domains and the corresponding nucleotides and recording the frequency of each nucleotide for each amino acid at positions 6 *or* 1′ (data not shown) as well as in the combination of 6 *and* 1′ (Figure S2A). The combinatorial search for generating figures S2A–C and S3 and for computing the nucleotide counts was performed under the technical computation environment MATLAB (www.mathworks.com) with a customized interface to the PPR and editing sites database under MS Excel.

**Figure 2:**
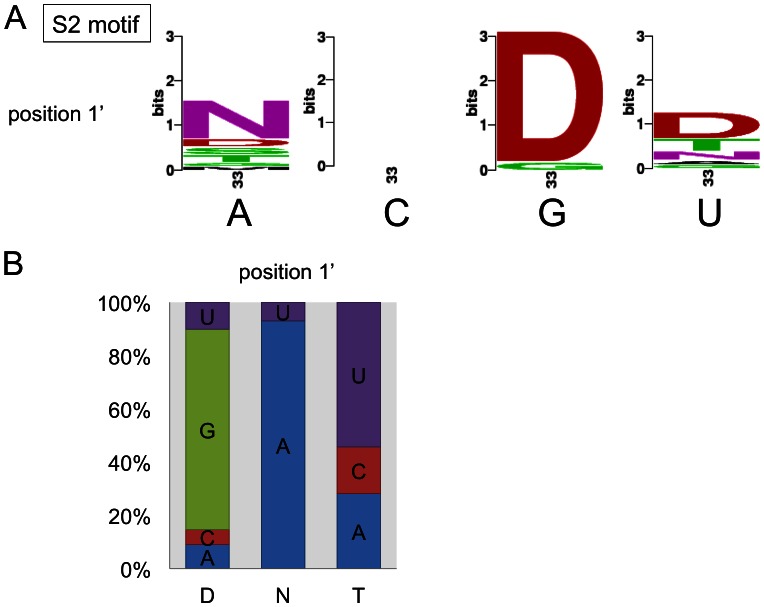
Amino acids in RNA editing PPR protein S2 motifs correlate with target nucleotides. (**A**) Sequence logos were constructed for each of the four nucleotides facing the respective S2 domains in the predicted PPR-RNA interaction at position –4 relative to the edited C (Figure 1). Coincidences between nucleotide and amino acid identities are seen for position 1′ (also labelled as amino acid 33). No coinciding amino acid preference is seen with the C nucleotide. (**B**) The amino acid identity at position 1′ shows the most prominent correlation between D (aspartic acid) and nucleotide G, N (asparagine) and A, T (threonine) and U. In the bar diagram, percentages of nucleotide identities coinciding with the respective amino acid are indicated. Nucleotide percentages are normalized by calculations with taking the A, C, G and U percentage into account as detailed in the methods section. Sequence logos were derived with the web-based software at weblogo.berkeley.edu [25].

**Figure 3:**
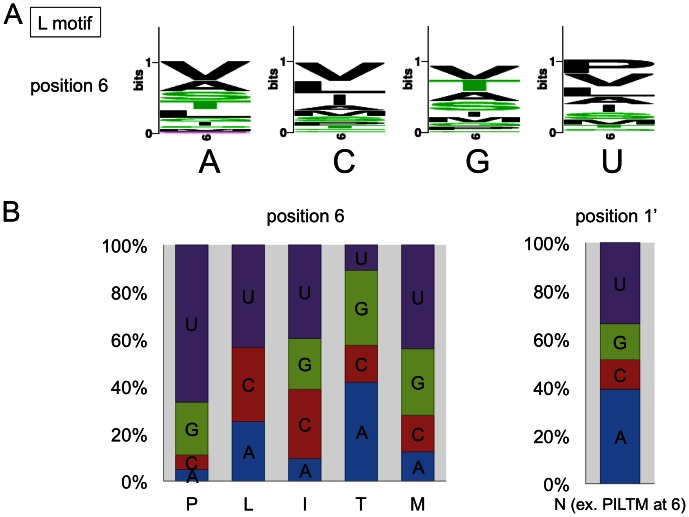
Amino acids at position 6 in RNA editing PPR protein L motifs correlate with nucleotide identities. (**A**) Sequence logos opposite each of the four nucleotides show the amino acid identities in L domains of predicted PPR-RNA interactions at position 6. Amino acid V (valine) is prominent at all nucleotide identities and thus possibly represents non-discriminatory spacer elements. (**B**) Correlations between amino acid identities at position 6 are most prominent for amino acid P and to a lower extent also for L, I, T and M with nucleotide U and amino acid T (threonine) with A or G. Position 1′ shows no discernible correlation when amino acids I, L, P, T or M are present at position 6. When these amino acid identities are excluded (ex.), a weak correlation can be seen with amino acid N to nucleotide identity A or U.

**Figure 4:**
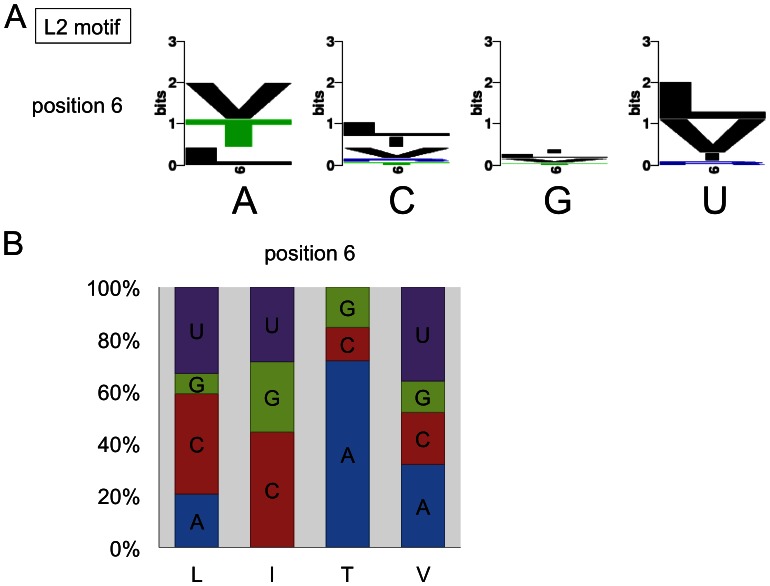
The L2 motifs in RNA editing PPR proteins correlate with nucleotide identities. (**A**) Correlations between amino acid and respective nucleotide identities in the target RNAs reveal preferential combinations with amino acid position 6 in the sequence logos. (**B**) Amino acid identities leucine and isoleucine at position 6 correlate with C, U or A (respectively G) in descending frequency, whereas threonine at this position is prevalent opposite nucleotide A. As in the L repeats, amino acid V occurs with any nucleotide. Preferences at position 1′ are not apparent in the sample size available.

To analyse the statistical probability, these data are compared to the overall number of nucleotides in the coding regions in all mRNAs in mitochondria and to those open reading frames in plastids where RNA editing has been observed (Figure S2B and Figure S2C). To avoid the effect of a biased nucleotide ratio in organellar transcripts, the computed ratios were further adjusted by correcting for the overall probability of a given nucleotide identity. For instance, the ratio of A in the listing (Figure S2B) is given by R(A) = N(A)/N(A+C+G+U) for each corresponding amino acid at the respective position, where N(·) denotes the respective nucleotide frequency. The probability of nucleotide A to occur at a certain position in organellar transcripts is given by NP(A) = N_total_(A)/N_total_(A+C+G+U). Adjusted nucleotide ratios AN(A) are deduced according to AN(A) = R(A)/NP(A) (Figure S2B and Figure S2C, and employed in Figures 1–4). Total nucleotide numbers are 12035 for A, 7634 for C, 8633 for G and 13724 for U. The resulting nucleotide probabilities (NP) are 0.286 for A, 0.182 for C, 0.205 for G and 0.327 for U. Furthermore, these adjusted nucleotide ratios are recalculated into percentages and then used as position-dependent scoring matrices in the program FIMO (link given below) for RNA editing site prediction (Fig. S2C).

The P-values for the actual nucleotide ratios to total nucleotide ratios were calculated using G-tests. If P<0.1 for at least one of four nucleotides, we handled the data as approved with sufficient significance for further analyses (Figure S2B and Figure S2C). The G-tests were calculated with an MS Excel spreadsheet downloaded from the web site of “Handbook of Biological Statistics” (http://udel.edu/~mcdonald/statgtestgof.html). Amino acid combinations or single amino acid identities at positions 6 and 1′ which occurred in fewer than three factors or less than eight incidences were not included in the further analysis.

### RNA Editing Site Prediction in the RNA

As previously surmised [8]–[10], we assumed that each PPR domain in a given PPR protein binds to one nucleotide and that the binding intensity of a PPR domain to a nucleotide correlates with the adjusted nucleotide ratio obtained by computational analysis. Therefore we directly employed the adjusted nucleotide ratio as putative binding intensity of each PPR domain and redefined this as “binding value”.

To predict the target RNA editing sites of a PPR protein, all PPR motifs in the PPR protein were aligned to the respective 5′ nucleotide sequences of all known RNA editing sites with the fourth nucleotide upstream of each editing site (nucleotide –4) assigned to the S2 motif. The binding values of each PPR motif domain were calculated with the FIMO program in the MEME suite (http://meme.nbcr.net/meme/fimo-intro.html) with 30 nucleotides upstream sequence of 34 chloroplast and 430 mitochondrial RNA editing sites in the respective coding regions (Figure S7). For mitochondrial transcriptomes, the sequences of all mitochondrial proteins with known functions and the ribosomal RNA sequences, and for chloroplast transcriptomes, all chloroplast proteins and ribosomal RNA sequences were retrieved from FLAGdb++ (http://urgv.evry.inra.fr/projects/FLAGdb/HTML/index.shtml) and used as reference sequence in the FIMO program. Predicted targets sites are ranked by p-value.

### RNA Editing Analysis of the MEF11 and MEF32 Mutants

The top 20 ranked RNA editing sites predicted by the FIMO program tool for the two E-class PPR proteins MEF11 and MEF32 were analysed experimentally. Total cellular RNA was purified from the respective T-DNA (MEF32 At4g14170: SALK_039629) or EMS mutants (*mef11-1*) [14] with the RNAeasy kit (Qiagen, Hilden, Germany). Reverse transcription reactions were performed with a 30-primers-set developed for mitochondrial transcripts in *Arabidopsis thaliana* [15]. PCR reactions were performed with the respective gene-specific primer sets. PCR products were purified by alkaline phosphatase and ExoI and were commercially sequenced (Macrogen, Seoul, Korea or LGC, Berlin, Germany).

## Results

Previously, amino acid positions 6 and 1′, respectively, in each PPR motif were noted to show correlations between amino acid identity and the presumably contacted nucleotide identity. These could function as discriminators to convey RNA sequence specificity depending on the order of the repeat elements in the respective PPR protein. Indeed, experimental evidence confirmed the influence of these amino acid positions [10]. Since the L, L2 and S2 type motifs characteristic for RNA editing PPR proteins could not be included, we here set out to determine their influence.

### The L, L2 and S2 Motifs Selectively Contact RNA Nucleotides

The recent computational analysis of the RNA sequence recognition code in PPR proteins by Kobayashi et al. [9] used 4614 PPR motifs from *Arabidopsis* PPR proteins. The subsequent improved analysis by Barkan et al. [10] is based upon the alignment of the P and S type repeats in three P-class PPR proteins and 37 E-class RNA editing factors. Here the L, S2 and L2 type motifs were not considered as RNA binding elements but as spacers between P and S motifs. This assignment is supported by the observation that these L, S2 and L2 elements display amino acid compositions very different from the P and S type repeats. According to the suggestion of Rivals et al. [16], S2 is the C-terminal repeat at the border of the PPR tract and the N-terminus of the E domain (Figure 1). The L2 element is located just upstream, N-terminal to the S2 motif. The L elements are spaced between the P and S type repeats, usually in the order P-L-S [16].

We reasoned that the L, S2 and L2 elements may also have to be considered as RNA contacts, since they may be required for RNA sequence specificity of short PPR proteins. Several RNA editing factors have only a very limited number of PPR motifs, e.g. MEF20 has only eight such repeats [17], MEF8 and MEF8S have only five including the L, L2 and S2 elements (Figure 1) [13]. If in e.g. MEF20 the L, L2 and S2 domains are not involved in nucleotide recognition, only four PPR repeats are left to specify target RNA editing sites. These could contact only four nucleotides in the one-on-one mode, which would not be sufficient to target a specific RNA editing site in plant mitochondria. In fact, these four nucleotides and the C at the editing position occur 31 times in the mitochondrial transcriptome.

Therefore at least these and possibly other L, L2 and S2 motifs should be considered to contribute to the sequence specific binding of the PPR proteins involved in RNA editing in plant organelles. Therefore we here probed these repeats to discern if they also show a nucleotide selective code. To identify potentially discriminatory amino acids in the L, L2 and S2 repeats, we performed a computational analysis of correlations between amino acid and corresponding nucleotide identities. As informational base we used 42 RNA editing PPR proteins for the analysis of potential amino acid-nucleotide correlations. These included two rice proteins with eight editing target sites, six *Physcomitrella* proteins with nine editing target sites and 34 *Arabidopsis* proteins with 57 editing target sites, a total of 74 RNA editing target sites. We focussed attention on the amino acid identities at the sites equivalent to the discriminatory positions in the P repeats [7]–[10].

### The Amino Acid at the 1′ Position of S2 Motifs Correlates with the Target Nucleotide Identity

The site-specific PPR RNA editing factors and the RNA target sequences show optimal correlations when the PPR domains are aligned 5′ to the editing sites starting from nucleotide position −4 relative to the edited C in the upstream direction [8]–[10]. The S2 motifs are accordingly positioned at the −4 nucleotides (Figure 1). To test for S2 nucleotide-amino acid correlations we probed 42 S2 domains against 74 nucleotide identities in the –4 position. Figure 2A shows in the sequence logos the frequencies of individual amino acid identities at position 33 (i.e. 1′) opposite either A, C, G or U nucleotides. Figure 2B depicts the reverse analysis and shows how often an A, C, G or U nucleotide is found opposite amino acid threonine (T), aspartic acid (D) or asparagine (N) at position 33 (i.e. 1′). These nucleotide coincidences were calculated after adjusting for the respective A, C, G and U contents of the mitochondrial and plastid coding sequences (see Methods). For example, opposite nucleotide G most often amino acid D is found in amino acid position 33 (Figure 2A). If amino acid N is in this position, usually nucleotide A is present in the RNA (Figure 2B and Figure S2).

This 33^rd^ amino acid position corresponds to the determining position 1′ of the degenerated P motifs in the N-terminal region of the extension (E) domain. This finding supports the observed weak similarity of the E domain with the structure of the PPR repeats and the interpretation that the E domain is a degenerated PPR motif.

The amino acid at position 6 is more variable in S2 than in P and S motifs and therefore requires a larger sample number than is presently available.

### In L and L2 Motifs Amino Acid Identities at Positions 6 or 1′ Correlate with Specific Nucleotides

Sequence logos constructed from 153 L motifs (without the L2 repeats) aligned with 258 target nucleotides show that at position 6 amino acids valine (V) and also alanine (A) are present opposite all four nucleotide identities (Figure 3A). This non-discriminating coincidence may reflect a frequent function of the L domain as spacer or placeholder that was suggested previously [9], [10].

Of the other position 6 amino acid identities, I, L, P, T and M correlate with a slight bias with different nucleotide identities. In the reverse analysis, most prominent are the absence of the G nucleotide opposite amino acid L and the positive correlation between amino acid P and nucleotide U (Figure 3B). In position 1′, a positive correlation is detected between amino acid N and nucleotides U or A. However, this is only seen when neither of the five biased amino acids I, L, P, T or M is present at position 6. That the presence of a 1′ amino acid to nucleotide correlation depends on the position 6 amino acid identity, may suggest that position 6 can overrule the influence of the amino acid identity at position 1′ at least in these instances. Here position 1′ refers to the amino acid identity at the first position of the next PPR element which is most often an S-type PPR.

Overall, the nucleotide - amino acid correlations in the L domains are much weaker than those in the P and S motifs. The potential significance of other amino acids at these positions remains unclear or undetectable due to the limited number of samples.

The L2 motif is the C-terminal L motif, located at the N-terminus of the S2 motif (Figure 1). The N-terminal region of the L2 motif, facing the ‘regular’ PPR tract, shows high similarity to the N-terminal region of ‘normal’ L motifs, but the C-terminus is rather different. This difference and the smaller sample number of only one L2 element per PPR protein complicate the correlative analysis. Sequence logos constructed from 42 L2 motifs aligned with 72 target nucleotides suggest that amino acids T, I, V and L at position 6 correlate with a selective nucleotide bias similar to the ‘normal’ L domains (Figure 4A). The reverse analysis, probing from the amino acid identity at position 6 the identity of the nucleotide opposite, shows biases for isoleucine, which is negatively correlated with nucleotide A and for threonine which is conspicuously absent opposite U but is positively correlated with A (Figure 4B and Figure S2).

### In P and S motifs Single Amino Acid Identities at Positions 6 or 1′ are Correlated with Nucleotide Preferences

The previously observed correlation between specific amino acid combinations at positions 6 and 1′ and nucleotide preferences [8]–[10] is confirmed by our computational analysis (Figure S1, Figure S2 and Figure S4). The major correlated amino acid pairs, T6N1’, T6D1’, N6D1’, N6N1’, N6S1’, N6T1’, S6N1’ and S6D1’, generally match the binding intensity between respectively modified repeats and corresponding RNA sequences demonstrated *in vitro* by Barkan et al. [10]. These typical amino acids combinations prevail in 85% of all P and S motifs.

**Figure 5:**
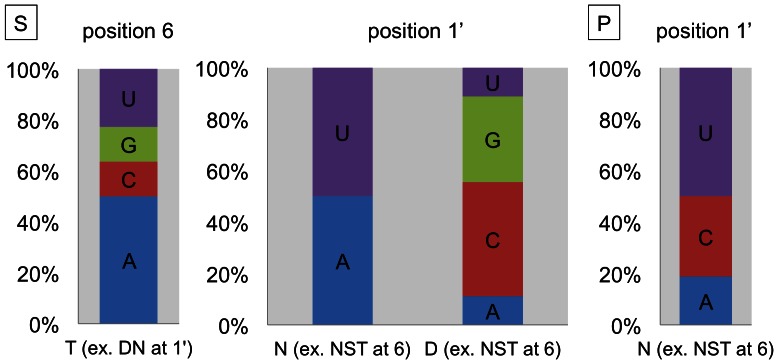
Positions 6 or 1′ in P and S motifs in RNA editing PPRs correlate with specific nucleotides. Depicted are individual connections of positions 6 or 1′ in those instances, where the most prominent combinatory amino acid identity correlations between positions 6 and 1′ are excluded as indicated (ex.). In these instances single amino acid positions correlate with distinct nucleotide preferences in S and P elements, respectively. For the S elements non-random distributions are found at positions 6 and 1′, for the P elements only at position 1′. The most prominent combinatory amino acid – nucleotide identity correlations which are excluded here have been identified previously (8–10).

To determine whether there are additional correlations beyond these combinations, we focused the next analyses on either of these two amino acid positions individually. Accordingly we scanned nucleotide - amino acid correlations for either position with excepting the prevalent amino acid at the respective other position 6 or 1′. This approach detects amino acid - nucleotide preferences in addition to the prevalent ones (Figure 5). For example, no C and G nucleotides are found opposite amino acid N at position 1′ in S motifs. While much less prominent than the combination of amino acid identities at positions 6 and 1′, these individual positions may prefer nucleotides singly or connected with rare amino acid identities at the respective other position 6 or 1′. To identify such additional rare combinations, yet larger sample numbers are required.

### Prediction of RNA Editing Target Sites

The computational analysis of the P, S, L, S2 and L2 motifs correlates nucleotide preferences with different combinations of amino acids at positions 6 and 1′ in all PPR elements. To evaluate the functional relevance of the correlations, we employed them to predict target nucleotide sequences from the respective amino acid identities in several PPR proteins known or suspected to be involved in RNA editing. The RNA sequences of editing sites were aligned starting from nucleotide –4 with the S2 element of the editing PPR protein. The number of the further upstream nucleotides is determined by the number of PPR motifs as in the previous studies [8]–[10]. We evaluated 430 RNA editing sites in *Arabidopsis* mitochondria coding regions and 34 sites in chloroplasts. Predicted binding intensities of each PPR-nucleotide pair were calculated as relative values, were compared between different RNA editing sites and used for the ranking as detailed in the methods section.

**Figure 6:**
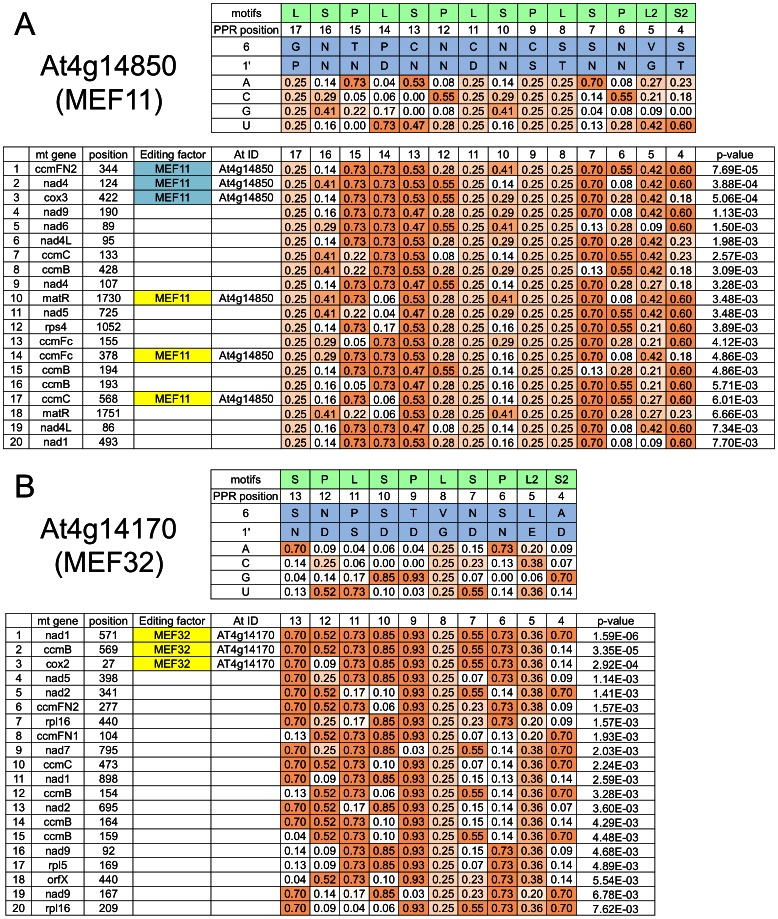
Prediction of nucleotide target sequences for MEF11 and the novel RNA editing PPR protein MEF32. (**A**) For MEF11 (At4g14850), the RNA editing sites ranked at positions 1, 2 and 3 out of 430 sites have been previously identified as target sites (shaded light blue) [17]. When we analysed all 20 top ranked editing sites in a MEF11 mutant, sites ranked 10, 14 and 17 turned out to be also targets of MEF11 (shaded yellow). (**B**) Target sequence predictions for the previously unassigned mitochondrial RNA editing factor encoded by At4g14170 are shown for the top ranked twenty sites. When we investigated these in a T-DNA mutant of the gene At4g14170, the three top ranked sites were identified as bona fide targets, at these nucleotides editing is absent in the mutant. This locus has been accordingly renamed to indicate that it codes for the novel RNA editing protein MEF32. The respective top parts in panels A and B show the PPR motifs considered (shaded light green; including the L2 and S2 elements) and their alignment to nucleotide positions which are counted 3′ to 5′ from the edited C (from right to left, −4 to −17 and −4 to −13, respectively). Amino acid identities at positions 6 and 1′ are given (shaded blue) and the respective scores are shown. In the box below, the locations of the top twenty ranked sites are indicated and the assigned specificity factor is given for experimentally confirmed targets, here MEF11 or MEF32. For each repeat the score at each site is given (shaded ocre with the color intensity reflecting the score) and the p-value of FIMO progaram as shown in the far right column is used for the ranking.

Predicted nucleotide binding intensities (binding value) for each PPR repeat in the editing protein MEF11 are shown in the upper part of Figure 6A. For example, the binding values of the P motif at position 15 (that is opposite nucleotide −15 of the edited nucleotide) are calculated to A = 0.73, C = 0.29, G = 0.41 and U = 0, respectively. To predict the target RNA editing sites for each PPR protein, the binding values of each of the individual PPR motifs are used as position-dependent scoring matrices for the FIMO program. This program converts log-odds scores for each of the RNA sequences into p-values, assuming a zero-order background model as detailed on the FIMO webpage (http://meme.nbcr.net/meme/fimo-intro.html). To predict the target sites of MEF11, the obtained p-values for all RNA editing sites are used for ranking the sites (Figure 6A, bottom part).

The accordingly top ranked RNA target motifs predicted for mitochondrial editing factor 11 (MEF11) include the editing sites where MEF11 is known to be involved at ranking positions 1, 2 and 3 [17]. The other top 20 ranked sites for MEF11 were analysed in the respective gene disrupted mutant plant. Of these predicted targets, *matR*-1730 at rank 10, *ccmFc*-378 (*ccb452*-378) at rank 14 and *ccmC*-568 (*ccb256*-568) at rank 17 were identified as previously overlooked targets of MEF11 (Figure S5). These sites had not been included in the original screen of mitochondrial editing sites in the MEF11 mutant.

In the next experimental test, we predicted the target RNA editing sites of the E-class PPR protein encoded by At4g14170, for which the target sites were unknown. To evaluate the predictive power of the program, we analysed a T-DNA insertion line of this gene for RNA editing defects at the top twenty predicted target sites (Figure 6B and Figure S5). In the respective cDNA of the T-DNA line of At4g14170, the three top ranked predicted target sites, *nad1*-571, *ccmB*-569 (*ccb206*-569) and *cox2*-27, are indeed unedited (Figure 6B and Figure S5), confirming the functional validity of the improved PPR prediction approach. The thus newly identified E-class PPR protein encoded by At4g14170 has now been renamed mitochondrial RNA editing factor MEF32.

### Inclusion of the L, L2 and S2 Motifs Improves Prediction of RNA Editing Target Sites

To evaluate if the inclusion of the L, L2 and S2 domains improves assignment and ranking of predicted target sites over the previous method of using only P and S elements, we compared the prediction of target sequences by the two approaches.

**Figure 7:**
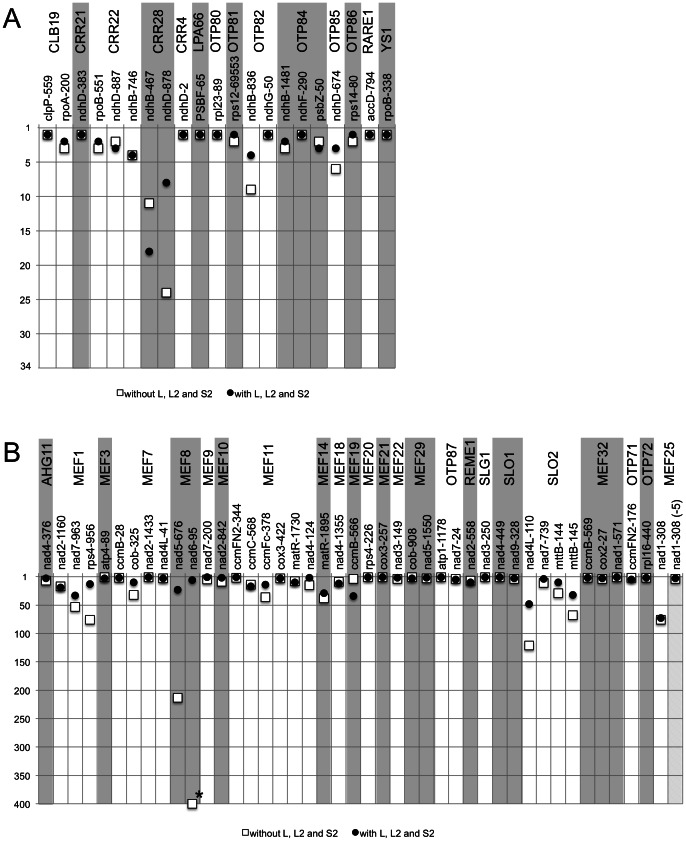
Inclusion of L, L2 and S2 repeats generally improves the prediction accuracy of RNA editing targets. Although the bona fide target sites are listed in the top ranks even without including the L, L2 and S2 repeats, their consideration mostly improves the prediction accuracy if only slightly. This suggests that these repeats also connect to target RNA sequences. Shown here are only the data for *Arabidopsis*. For *Physcomitrella* mitochondria, prediction ranks target sites always at the top, but then there are only very few editing sites in this moss (Figure S3). (**A**) Prediction of the target sites for the known chloroplast editing factors finds the identified targets within the top ranks out of the 34 RNA editing sites in chloroplasts of *Arabidopsis*. Prediction from only the P and S repeats (□) is usually sufficient, but inclusion of the L, L2 and S2 elements (•) often improves the ranking. (**B**) Analogous improvements of the predictions are seen within the 430 editing sites considered for mitochondrial PPR proteins. In a few instances the predicted PPR-RNA interaction drops in rank when the L, L2 and S2 elements are included (e.g. the targets of MEF18 and MEF19; further details are given in Figure S3). The *nad6*-95 target site of MEF8 (asterisk) cannot be ranked since the p-value is >1 in the FIMO program evaluation.

Figure 7 shows the rankings of the predicted RNA editing target sites of the presently published RNA editing factors as listed in Figure S3. Both approaches rank the known target sites for each RNA editing factor in the upper 50% in plastids (Figure 7A) as well as in mitochondria (Figure 7B). However, the ranking of many sites improves considerably when the L, L2 and S2 motifs are included. For example, one the two experimentally identified target sites of CLB19, *rpoA*-200, is predicted at position three of 34 plastid editing sites when only the P and S codes are used (Figure 7A). Inclusion of the L, L2 and S2 elements increases the prediction of this site to rank two. Overall, in plastids, the predictions of nine target sites improve by inclusion of the L, L2 and S2 motifs, eleven target sites are equally well predicted without these motifs, and three predictions are better with only the P and S elements.

In the mitochondrial editing factors, overall target site prediction with the L, L2 and S2 elements improves in 19 instances, nine target sites are equally ranked without these motifs, and nine predictions are better with only the P and S elements (Figure 7B). Improved ranking is most striking with the short PPR proteins such as MEF8 which contain only few P and S elements. Another short protein, MEF20, predicts five candidate target sites with equal binding values from the P and S repeats, while consideration of the L, L2 and S2 motifs yields a clear ranking and selects the actual MEF20 target sites to the top (Figure S3). The ranking improvement increases when the entire transcriptomes of chloroplasts or mitochondria are used as reference sequences (Figure S7). Inclusion of the L, L2 and S2 motifs in the prediction thus generally improves the specificity towards the target RNA editing sites over the consideration of only the P and S motifs.

## Discussion

### Amino Acid Identities in L Motifs Correlate with Nucleotide Preferences in the RNA

The detailed analysis shows that the structures of many L and L2 motifs show a correlation with the corresponding nucleotides in the target RNA. However, the nucleotide bias in these L type motifs is less pronounced than that of the P and S elements and is limited to few specific amino acid identities at positions 6 and/or 1′ in about half of the L and L2 elements. About 45% of the L and L2 motifs do not show any nucleotide preference. These L and L2 motifs may actually function as spacers between P and S motifs as proposed by Barkan et al. [10]. In our analyses we did not include non-edited cytidines which have to be discriminated against by the PPR RNA editing factors. The low but detectable increase in the specificity of the RNA editing factors by inclusion of the L motifs may be important if not necessary to distinguish RNA editing sites from other not-to-be-edited cytidines in organellar transcripts.

The importance of the L motif for the function of the RNA editing PPR proteins is supported by the effect of the *MEF3* SNP mutation in ecotype L*er* in comparison to the Col accession. The only two unique amino acid exchanges between the two ecotypes are located in one of the L domains in MEF3. These differences must be responsible for the lower level of RNA editing at the *atp4*-89 target site in ecotype L*er* (50%) in comparison to the 100% in Col [18]. One of these two amino acid alterations occurs at position 1′. The substitution of amino acid D by N from Col to L*er* changes the nucleotide preference from ‘neutral’ to ‘G negative’ in our prediction. This shift may lead to a PPR-nucleotide mismatch and may thus decrease the overall binding intensity to the target RNA sequence and consequently also result in the observed lower RNA editing efficiency of this site in L*er* plants.

### Comparison of the Nucleotide Recognition Patterns of P, S and L Elements

The amino acids at positions 6 and 1′ in P and S and here also in L motifs are correlated with nucleotide identities and have consequently been proposed to be potential nucleotide binding amino acids [8]–[10]. In our analysis, the P and S motifs slightly differ in their bias of amino acid combinations and corresponding nucleotide identities. Most prominently, the amino acid combination NN at positions 6 and 1′ clearly shows a preference for C and U nucleotides in the P motifs but not in the S type repeats (Figure S2 and Figure S4).

Different from both P and S motifs are the amino acid – nucleotide correlations in the L elements. Among the four amino acid moieties at position 6 in the L domains for which we find nucleotide preferences, threonine in P and S domains has been correlated with A and G nucleotides. Other prevalent amino acids at position 6, notably L, I, M and P, so far have not been reported to be able to act as nucleotide binding amino acids, however any amino acid in a peptide chain is potentially able to attach to any nucleotide [19]. Alternatively, these four amino acids may be merely tolerated by the contacted nucleotide. The lower nucleotide to amino acid correlation bias observed in L motifs in comparison to that of the P and S motifs may result from such a non-biased weak nucleotide affinity.

### Evaluation of the Prediction Accuracy

The inclusion of the L, L2 and S2 repeats generally improves the correlation between amino acid identities in the PPR repeats and the target sequences in the RNA. This improvement is seen in the better accuracy in the prediction of these target sites from the PPR structures (Figure 7 and Figure S3). The high success rate of predicting correct target sites within the top twenty is by no means perfect, but may be better than it seems. Further of the high-scoring target sites may be genuine targets even though they are still edited in mutant lines of the respective RNA editing PPR protein. A target site may be hidden in a mutant when another PPR protein compensates for the missing factor and sustains RNA editing. This has been documented for the MEF8 and MEF8S PPR proteins [13].

Some experimentally identified RNA editing target sites are ranked rather low in the prediction. Several possible explanations can be considered why e.g. predictions from MEF14 or MEF1 do not match the target sites very well. Firstly, amino acid identities at other positions than 6 and 1′ may influence the binding preference. Some of the EMS induced mutations and of the SNPs in ecotypes of *Arabidopsis* with lower RNA editing (e.g. MEF1 in ecotype L*er*) occur at other positions within PPR motifs. These variant amino acids may influence the binding specificity and alter the RNA sequence recognized.

Secondly, the E and/or DYW domains may influence the target RNA sequence pattern. More than 90% of the −1 nucleotides at editing sites are C or U [20], this nucleotide locating near the E and/or DYW domains of the specific PPR protein. Furthermore, the E domains display PPR like features and likely evolved from another PPR repeat.

Thirdly, the distance between the RNA editing site and the binding site of the cognate PPR protein may vary. As in the previous analyses [8]–[10], we aligned the PPR elements from the −4 position of the respective RNA editing site. This appears to be correct in most instances, but there are exceptions. For example, two successive editing sites are recognized by the PPR protein SLO2 [21] which suggests that the distance between the PPR protein and the RNA editing site can be flexible in some interactions. The recently identified MEF25 [22], which is necessary for one of the two successive RNA editing sites, matches the target sequence much better with an alignment from nucleotide –5, which improves ranking of the target site from position 73 to position 3 (Fig. S3). Nevertheless, most of the target predictions are optimal for the –4 alignment when we probed alternative shifted alignments. For several PPR proteins the distance to the edited nucleotide is very rigid. For example, the CRR28 and MEF11 factors cannot edit the C-nucleotide immediately upstream the bona fide editing site. MEF20 cannot alter the C subsequent to its respective target nucleotide and CRR22 precisely targets the central of three consecutive C-nucleotides [11], [14], [23].

Fourthly, unique or very rare amino acid combinations at positions 6 and 1′ or at other positions may specify a nucleotide preference. Such correlations can only be identified in much larger numbers of samples than presently available.

### Do RNA Editing Factors with Many PPR Repeats Allow Gaps in the Contact to RNA?

Previous analyses of coincidences between amino acids in P-type PPR proteins and RNA nucleotide identities had allowed gaps of one or two non-binding repeats opposite the respective nucleotides [8]–[10]. Experimental evidence suggests that P-class PPR proteins with large numbers of PPR elements can bind to their target sequence with several mismatched nucleotide - PPR element gaps or loop-outs. Since so far there is no experimental evidence that E-class PPR proteins permit analogous gaps, we did not allow for loop-outs of nucleotides in the RNA or of repeat elements in the PPR proteins. In the resulting alignments the rare scores lower than 0.05 potentially derive from analogous gaps between protein and RNA (Figure S3). This will have to be investigated experimentally.

Contrary to P-type PPR proteins, binding of the C-terminal repeats should be important for the E and DYW-class PPR proteins, since the RNA editing site is always positioned at the C-terminal end of the respective PPR protein. One of the shortest specific RNA editing factors, MEF20, possesses only eight PPR domains which by default have to be sufficient for specific targeting. By extrapolation, the eight C-terminal PPR domains may be sufficient for specific targeting in at least some other RNA editing factors as well. To investigate this possibility, we compared the ranking of target editing sites between predictions derived from consideration of all PPR motifs and and predictions which included only the eight PPR motifs at the respective C-termini (Figure S6). Half of the PPR editing factors still ranked in the top 10%, including all those with more than 20 PPR repeats. Interestingly, MEF14 and SLO2 actually improve ranking of their target sites when considering only the eight C-terminal PPR motifs suggesting that the rest of the PPR motifs, located towards the N-terminus, may not contribute to the specific targeting.

While this report was under review, an analysis was published which suggests that a third amino acid position may be involved in determining the nucleotide identity bound by a given repeat element in RNA editing PPR proteins [24]. In our analysis we did not observe such an additional discriminating position which may be due to the differing approaches and selections.

### Conclusions

The correlative analysis of the L, L2 and S2 type repeats shows an analogous albeit weaker connection to the respective nucleotide identities than the P and S elements and suggests that these repeats often also contact the RNA. Inclusion of the L, L2 and S2 type repeat correlations generally improves the prediction accuracy of finding target RNA sequences for a given E-class RNA editing PPR protein. This will increase the efficiency to assign RNA editing target sites to novel E-class PPR proteins.

The improved correlation will furthermore enhance the chances of manipulating these RNA editing PPR proteins. For example, it should be easier to complement (or abolish) only one of multiple RNA editing targets by respective specific alterations in the PPR protein. This will give access to analyse the effect of an individual RNA editing event even when the complete loss of the editing factor is lethal.

Finally, this information will allow to create RNA editing factors which can edit any cytidine in any transcript specifically and thus facilitate the generation of ‘RNA mutants’ in mitochondria. Such constructs will circumvent the difficulties encountered in mitochondrial transformation. These manipulations are not restricted to plants or to mitochondria, but PPR proteins can be generated for any RNA target in any organism. Especially the here analysed type of E-class PPR proteins will be very interesting since during editing they contact the RNA and then dissociate again. Pure P-type proteins often bind tightly to their RNA target and cannot be removed.

## Supporting Information

Figure S1 **Evaluation of coincidences between amino acids within PPR elements and corresponding nucleotides.** Part of the entire set of aligments is shown for the PPR editing protein MEF1. Each amino acid in each motif is analysed for co-occurences between amino acid and corresponding nucleotide identities. Amino acids at positions 1′ and 6 show the strongest correlations as depicted in figure 1. For the length of motif S2 convention assigns 36 nucleotides which positions amino acid 1′ into S2 (indicated by an arrow and shading), different from all other elements where amino acid 1′ is usually assigned to the first amino acid position of the C-terminally adjacent element.
(PDF)

Figure S2 **(A) Printout of an example from the MATLAB output alignment.** In this sample, amino acids at position 6 (top horizontal column) with each of the four nucleotides (second horizontal column) are correlated with the amino acids present at position 1′ (left vertical column). The numbers of appearances in the 41 PPR RNA editing proteins are given in the figure. This and further data sets are evaluated and compiled in Figure S2B. **(B)** **The data set used for assigning co-occurences between nucleotides in target sequences and amino acid identities at positions 6 and/or 1′ in the indicated motifs of RNA editing PPR proteins.** For example, for the S2 motif, amino acid D is found at position 1′ four times correlated with an A nucleotide identity, two times with C, 22 times with G and seven times with U. The respective probabilities (P-value) calculated by G-test for each nucleotide are given color coded in the right columns, the color code is shown on the right bottom. **(C)** **The adjusted data set used for assigning co-occurences at positions 6 and/or 1′ in the indicated motifs of RNA editing PPR proteins.** The raw numbers of nucleotide-amino acid co-occurrences from figure S2B were adjusted for the G+C content of mitochondrial sequences. These adjusted values recalculated as total ratios were used in the further analyses.
(PDF)

Figure S3 **The data set used for** **figure 6** **as derived with the prediction tool shows the nucleotide target sequences assigned to RNA editing PPR proteins based upon their amino acid identities at positions 6 and 1′.** In the upper left, the gene names and their identifier numbers are given, and species, type of PPR protein (E or DYW), the organellar locations and the references as listed in References S1 are indicated. Below, the respective target sites are identified by gene name and the nucleotide position affected. For each of the PPR repeats, which are displayed from right to left in the C- to N-terminal direction, the p-values and the ranking of the respective target sites aligned from nucleotide –4 of the respective target sequence are given with or without considering the L, L2 and S2 elements as indicated. For MEF8 the predicted target sites are found several times in the mitochondrial set of editing sites, this and the low ranking is due to the small number of PPR elements in this protein. In the upper part, data for *Arabidopsis thaliana* (At) are shown with the plastid factors shaded green, in the lower part predictions for the PPR proteins for editing in mitochondria of *Physcomitrella patens* (Pp) are listed, shaded light green. At the bottom, predictions for *Oryza sativa* (Os) are given shaded yellow with the genuine target sequence put into the *Arabidopsis* editing site set (with At). Red cells in the target sequence indicate the nucleotide encoded as C in the genome and changed to U by RNA editing. Five of the seven targets of OGR1 are not found in *Arabidopsis*. Prediction for the rice editing site *ccmC*-458 is reasonable with the genomic sequence (unedited; rank 15), but deteriorates down to rank 71 after another site in the upstream sequence is edited, i.e. converted to U in the target motif. Not included in the calculations were the here identified MEF32, the new target sites of MEF11 and the PPR proteins OTP71, OTP72 and MEF25 which became avaliable after we had initiated the assignments.
(PDF)

Figure S4 **Correlation between amino acid combinations at positions 6 and 1′ in P and S motifs and nucleotide identities.** The amino acid combinations are given in the order 6 and 1′. Displayed are the percentages of coincidences between a given amino acid combination and the nucleotide identity. Amino acid combinations are shown from left to right ordered by their number of occurrence. These data are compiled from data as shown in figure S2C. For the P-elements the combinations N6D1’, N6N1’, T6N1’, T6D1’, N6T1’, N6S1’, S6N1’ and S6D1’ show the strongest correlations.
(PDF)

Figure S5 **Target sites predicted for MEF11 and MEF32 are analysed in respective mutant plants.** The top panels show a comparison of the cDNA sequences at the new target sites predicted for MEF11 between Col-0 wild type plants and the knock-out mutant *mef11*-1. While in the wild type plants the genomic encoded C is changed to T in the cDNA, the C remains unedited in mutant *mef11*-1. Site *ccmFc*-378 (*ccb452*-378) is a silent nucleotide exchange and is edited to only about 40% in Col-0 wild type plants. The lower panels show a comparison between the cDNA sequences at the target sites predicted for the previously unassigned PPR RNA editing factor MEF32 between Col-0 wild type plants and the knock-out mutant *mef32*. While in the wild type plants the genomic encoded C is changed to T in the cDNA, the C remains unedited in the mutant. The arrow points to the C peak not present in the wild type plants.
(PDF)

Figure S6 **The eight C-terminal PPR elements including the L, L2 and S2 repeats are often sufficient to predict RNA editing targets.** (**A**) Prediction of the target sites for the known chloroplast editing factors of *Arabidopsis* identifies bona fide targets within the top ranks from only the eight PPR elements at the C-terminus of the respective protein. Actually the ranking within the 34 RNA editing sites in chloroplasts is often better with only these eight PPR elements than when all PPR elements are included. (**B**) Analogous comparative analysis of the predictions within 430 editing sites considered for mitochondrial PPR proteins usually shows less faithful ranking with only the eight PPR elements at the C-terminus of the respective protein. Only in a few instances such as MEF14 and some sites of SLO2 the predicted PPR-RNA interaction is increased in rank in comparison to the prediction from all PPR elements.
(PDF)

Figure S7 **Inclusion of L, L2 and S2 repeats generally improves the prediction accuracy of RNA editing targets in the respective entire transcriptome.** The bona fide RNA editing target sites will have to be identified in vivo by the PPR protein factor against the presence of all C nucleotides in the respective organelle. Screening the prediction accuracy within all C nucleotides with or without including the L, L2 and S2 repeats, their inclusion generally improves the ranking considerably. Shown here are data for selected RNA editing PPR proteins for plastids and mitochondria (MEFs) from *Arabidopsis*. Screening was done against all C nucleotides in all transcripts in plastids (17.886) and in the mitochondrial transcripts with known functions respectively, both as annotated in the Flagdb. Changes in the ranking predictions are seen for example with the novel mitochondrial PPR protein MEF32 for which rankings change from positions 5 to 3, from 61 to 18 and from 3 to 1 upon inclusion of the L, L2 and S2 repeats. For MEF11, the predicted PPR-RNA interactions change rank from positions 92 to 4, from 295 to 331, from 100 to 27, from 209 to 184 and from 342 to 21 when the L, L2 and S2 elements are included. Some target sites (asterisks) are not ranked in the top 1000.
(PDF)

References S1 (DOCX)
