# Supplementary material for: Improved Computational Target Site Prediction for Pentatricopeptide Repeat RNA Editing Factors
Source: PLoS One. 2013 Jun 6;8(6):e65343. doi: 10.1371/journal.pone.0065343 (PMC3675099; doi:10.1371/journal.pone.0065343)
Supplement: Figure S2 — (A) Printout of an example from the MATLAB output alignment. In this sample, amino acids at position 6 (top horizontal column) with each of the four nucleotides (second horizontal column) are correlated with the amino acids present at position 1′ (left vertical column). The numbers of appearances in the 41 PPR RNA editing proteins are given in the figure. This and further data sets are evaluated and compiled in Figure S2B. (B) The data set used for assigning co-occurences between nucleotides in target sequences and amino acid identities at positions 6 and/or 1′ in the indicated motifs of RNA editing PPR proteins. For example, for the S2 motif, amino acid D is found at position 1′ four times correlated with an A nucleotide identity, two times with C, 22 times with G and seven times with U. The respective probabilities (P-value) calculated by G-test for each nucleotide are given color coded in the right columns, the color code is shown on the right bottom. (C) The adjusted data set used for assigning co-occurences at positions 6 and/or 1′ in the indicated motifs of RNA editing PPR proteins. The raw numbers of nucleotide-amino acid co-occurrences from figure S2B were adjusted for the G+C content of mitochondrial sequences. These adjusted values recalculated as total ratios were used in the further analyses. (PDF) [file pone.0065343.s002.pdf]

S motifs position 6 and positon 1’

position 6

position 1’

	A	A	A	A	C	C	C	C	D	D	D	D	E	E	E	E	F	F	F	F
	A	C	G	U	A	C	G	U	A	C	G	U	A	C	G	U	A	C	G	U
A																				
C																				
D			1	1			1													
E																				
F																				
G																				
H	1																			
I																				
K																				
L																				
M																				
N	2			1	3															
P	1																			
Q																				
R		2																		
S																				
T				1						1										
V																				
W																				
Y																				

	G	G	G	G	H	H	H	H	I	I	I	I	K	K	K	K	L	L	L	L
	A	C	G	U	A	C	G	U	A	C	G	U	A	C	G	U	A	C	G	U
A																				
C																				
D														2	1		1	2		
E																				
F																				
G																				
H																				
I																				
K																				
L																				
M																				
N								2				1								
P																				
Q																				
R																				
S	2																			
T				1																
V																				
W																				
Y																				

	M	M	M	M	N	N	N	N	P	P	P	P	Q	Q	Q	Q	R	R	R	R
	A	C	G	U	A	C	G	U	A	C	G	U	A	C	G	U	A	C	G	U
A																				
C											1									
D					12	12	4	51												
E						1														
F																				
G								1												
H								1												
I																				
K																				
L																				
M																				
N					6	8	13	8				1								
P																				
Q																				
R																				
S					2	5	1	5												
T						15	3	3												
V																				
W																				
Y																				

	S	S	S	S	T	T	T	T	V	V	V	V	W	W	W	W	Y	Y	Y	Y
	A	C	G	U	A	C	G	U	A	C	G	U	A	C	G	U	A	C	G	U
A																				
C																				
D	1		11	2	4	2	23	5												
E	1				1															
F																				
G										1										
H					1		2													
I																				
K	2		1		1															
L	1				3			1												
M																				
N	24	3	1	5	18	1	3	5												
P																				
Q								2												
R																				
S				1	3	3	1	2				1								
T					2															
V																				
W																				
Y																				

Figure S2A

	Nucleotides occurrence				P-value (G-test)			
S2 motif								
position 33 (1')	A	C	G	U	A	C	G	U
**D**	4	2	22	7	1.40E-02	2.98E-02	5.87E-08	9.56E-02
**N**	17	0	0	2	2.50E-08	5.79E-03	3.12E-03	2.35E-02
**T**	2	1	0	6	6.62E-01	5.60E-01	4.19E-02	3.71E-02

L2 motif								
position 6	A	C	G	U	A	C	G	U
**L**	5	6	1	10	5.30E-01	2.94E-01	3.04E-02	2.13E-01
**I**	0	4	2	5	6.45E-03	1.54E-01	8.44E-01	3.78E-01
**T**	7	1	1	0	2.27E-03	5.60E-01	4.52E-01	7.64E-03
**V**	8	4	2	14	9.94E-01	5.83E-01	4.90E-02	5.82E-02

L motif								
position 6	A	C	G	U	A	C	G	U
**P**	1	1	3	21	9.68E-04	2.60E-02	2.24E-01	4.46E-07
**L**	6	6	0	16	3.85E-01	6.61E-01	3.34E-04	7.91E-03
**I**	2	5	3	13	1.82E-02	6.64E-01	3.48E-01	1.89E-02
**T**	10	3	5	4	9.49E-02	5.68E-01	8.02E-01	1.27E-01
**M**	2	2	3	11	7.19E-02	4.10E-01	6.76E-01	1.35E-02

L motif								
position 1'	A	C	G	U	A	C	G	U
**N (ex. PILTM at 6)**	12	3	3	16	4.00E-01	1.24E-01	6.30E-02	8.19E-02

S motif								
position 6	A	C	G	U	A	C	G	U
**T (ex. DN at 1')**	11	3	3	5	3.53E-02	5.68E-01	4.00E-01	3.05E-01

S motif								
position 1'	A	C	G	U	A	C	G	U
**N (ex. NST at 6)**	5	0	0	5	1.56E-01	4.53E-02	3.20E-02	2.58E-01
**D (ex. NST at 6)**	1	4	3	1	2.03E-01	7.00E-02	3.72E-01	1.31E-01

P motif								
position 1'	A	C	G	U	A	C	G	U
**N (ex. NST at 6)**	3	5	0	8	3.62E-01	2.07E-01	6.68E-03	1.52E-01

		Nucleotides occurrence				P-value (G-test)			
S motif									
position 6	position 1'	A	C	G	U	A	C	G	U
**N**	**D**	12	12	4	51	4.94E-03	4.83E-01	8.37E-05	7.12E-09
**N**	**N**	6	8	13	8	1.14E-01	4.85E-01	2.42E-02	2.02E-01
**T**	**D**	4	2	23	5	1.78E-02	3.52E-02	3.26E-09	1.67E-02
**S**	**N**	24	3	1	5	1.66E-07	1.42E-01	2.83E-03	2.19E-02
**T**	**N**	18	1	3	5	4.36E-05	2.16E-02	1.93E-01	9.97E-02
**N**	**T**	0	15	3	3	1.67E-04	9.72E-08	4.59E-01	5.37E-02
**S**	**D**	1	0	11	2	4.37E-02	1.78E-02	3.29E-06	1.15E-01
**N**	**S**	2	5	1	5	2.61E-01	8.64E-02	2.01E-01	6.60E-01

P motif									
position 6	position 1'	A	C	G	U	A	C	G	U
**N**	**D**	10	17	11	64	1.41E-05	7.01E-01	1.85E-02	8.17E-09
**N**	**N**	7	29	5	26	2.93E-04	2.16E-06	3.08E-03	2.90E-01
**T**	**N**	23	1	5	0	1.56E-08	1.48E-02	6.53E-01	1.69E-06
**T**	**D**	1	0	18	1	6.60E-03	4.64E-03	2.11E-11	2.27E-03
**N**	**T**	1	10	0	7	1.24E-02	3.89E-04	4.02E-03	5.78E-01
**N**	**S**	1	12	0	2	3.20E-02	1.92E-07	8.63E-03	8.46E-02
**S**	**N**	9	1	0	2	8.95E-04	3.33E-01	1.88E-02	2.10E-01
**S**	**D**	2	4	6	2	2.04E-01	3.42E-01	5.96E-02	1.15E-01

P<0.05	positive
0.05<P<0.1	positive
0.05<P<0.1	negative
P<0.05	negative

Figure S2B

	Corrected ratio					Ratio in total			
S2 motif									
position 33 (1')	A	C	G	U		A	C	G	U
**D**	0.40	0.31	3.06	0.61		0.09	0.07	0.70	0.14
**N**	3.12	0.00	0.00	0.32		0.91	0.00	0.00	0.09
**T**	0.78	0.61	0.00	2.04		0.23	0.18	0.00	0.60

L2 motif									
position 6	A	C	G	U		A	C	G	U
**L**	0.79	1.50	0.22	1.39		0.20	0.38	0.06	0.36
**I**	0.00	2.00	0.89	1.39		0.00	0.47	0.21	0.33
**T**	2.72	0.61	0.54	0.00		0.70	0.16	0.14	0.00
**V**	1.00	0.79	0.35	1.53		0.27	0.21	0.09	0.42

L motif									
position 6	A	C	G	U		A	C	G	U
**P**	0.13	0.21	0.56	2.47		0.04	0.06	0.17	0.73
**L**	0.75	1.18	0.00	1.75		0.20	0.32	0.00	0.48
**I**	0.30	1.20	0.63	1.73		0.08	0.31	0.16	0.45
**T**	1.59	0.75	1.11	0.56		0.40	0.19	0.28	0.14
**M**	0.39	0.61	0.81	1.87		0.11	0.17	0.22	0.51

L motif									
position 1'	A	C	G	U		A	C	G	U
**N (ex. PILTM at 6)**	1.23	0.49	0.43	1.44		0.34	0.14	0.12	0.40

S motif									
position 6	A	C	G	U		A	C	G	U
**T (ex. DN at 1')**	1.75	0.75	0.66	0.70		0.45	0.19	0.17	0.18

S motif									
position 1'	A	C	G	U		A	C	G	U
**N (ex. NST at 6)**	1.75	0.00	0.00	1.53		0.53	0.00	0.00	0.47
**D (ex. NST at 6)**	0.39	2.45	1.62	0.34		0.08	0.51	0.34	0.07

P motif									
position 1'	A	C	G	U		A	C	G	U
**N (ex. NST at 6)**	0.65	1.72	0.00	1.53		0.17	0.44	0.00	0.39

		Corrected ratio					Ratio in total			
S motif										
position 6	position 1'	A	C	G	U		A	C	G	U
**N**	**D**	0.53	0.84	0.25	1.98		0.15	0.23	0.07	0.55
**N**	**N**	0.60	1.26	1.81	0.70		0.14	0.29	0.41	0.16
**T**	**D**	0.41	0.32	3.29	0.45		0.09	0.07	0.74	0.10
**S**	**N**	2.54	0.50	0.15	0.46		0.70	0.14	0.04	0.13
**T**	**N**	2.33	0.20	0.54	0.57		0.64	0.06	0.15	0.16
**N**	**T**	0.00	3.93	0.70	0.44		0.00	0.78	0.14	0.09
**S**	**D**	0.25	0.00	3.82	0.44		0.06	0.00	0.85	0.10
**N**	**S**	0.54	2.12	0.37	1.18		0.13	0.50	0.09	0.28

P motif										
position 6	position 1'	A	C	G	U		A	C	G	U
**N**	**D**	0.34	0.92	0.52	1.92		0.09	0.25	0.14	0.52
**N**	**N**	0.36	2.38	0.36	1.19		0.08	0.55	0.08	0.28
**T**	**N**	2.77	0.19	0.84	0.00		0.73	0.05	0.22	0.00
**T**	**D**	0.17	0.00	4.38	0.15		0.04	0.00	0.93	0.03
**N**	**T**	0.19	3.06	0.00	1.19		0.04	0.69	0.00	0.27
**N**	**S**	0.23	4.40	0.00	0.41		0.05	0.87	0.00	0.08
**S**	**N**	2.62	0.46	0.00	0.51		0.73	0.13	0.00	0.14
**S**	**D**	0.50	1.57	2.09	0.44		0.11	0.34	0.45	0.10

Figure S2C
