# Supplementary material for: Improved Computational Target Site Prediction for Pentatricopeptide Repeat RNA Editing Factors
Source: PLoS One. 2013 Jun 6;8(6):e65343. doi: 10.1371/journal.pone.0065343 (PMC3675099; doi:10.1371/journal.pone.0065343)
Supplement: Figure S3 — The data set used for figure 6 as derived with the prediction tool shows the nucleotide target sequences assigned to RNA editing PPR proteins based upon their amino acid identities at positions 6 and 1′. In the upper left, the gene names and their identifier numbers are given, and species, type of PPR protein (E or DYW), the organellar locations and the references as listed in References S1 are indicated. Below, the respective target sites are identified by gene name and the nucleotide position affected. For each of the PPR repeats, which are displayed from right to left in the C- to N-terminal direction, the p-values and the ranking of the respective target sites aligned from nucleotide –4 of the respective target sequence are given with or without considering the L, L2 and S2 elements as indicated. For MEF8 the predicted target sites are found several times in the mitochondrial set of editing sites, this and the low ranking is due to the small number of PPR elements in this protein. In the upper part, data for Arabidopsis thaliana (At) are shown with the plastid factors shaded green, in the lower part predictions for the PPR proteins for editing in mitochondria of Physcomitrella patens (Pp) are listed, shaded light green. At the bottom, predictions for Oryza sativa (Os) are given shaded yellow with the genuine target sequence put into the Arabidopsis editing site set (with At). Red cells in the target sequence indicate the nucleotide encoded as C in the genome and changed to U by RNA editing. Five of the seven targets of OGR1 are not found in Arabidopsis. Prediction for the rice editing site ccmC-458 is reasonable with the genomic sequence (unedited; rank 15), but deteriorates down to rank 71 after another site in the upstream sequence is edited, i.e. converted to U in the target motif. Not included in the calculations were the here identified MEF32, the new target sites of MEF11 and the PPR proteins OTP71, OTP72 and MEF25 which became avaliable after we had initi [file pone.0065343.s003.pdf]

		PPR position	26	25	24	23	22	21	20	19	18	17	16	15	14	13	12	11	10	9	8	7	6	5	4	3	2	1	P-value	Rank without L, L2 and S2	P-value	Rank with L, L2 and S2
**Gene name**	CLB19	motifs																	P	L	S	S	P	L	S	P	L2	S2				
**AGI number**	At1g05750	6																	T	I	T	N	T	I	N	N	T	G				
**Type**	E	1'																	N	H	N	D	D	N	T	D	R	N				
**Species**	At	A																	0.73	0.08	0.64	0.15	0.04	0.08	0.00	0.09	0.70	0.91				
**Localization**	cp	C																	0.05	0.31	0.06	0.23	0.00	0.31	0.78	0.25	0.16	0.00				
**ref.**	1	G																	0.22	0.16	0.15	0.07	0.93	0.16	0.14	0.14	0.14	0.00				
		U																	0.00	0.45	0.16	0.55	0.03	0.45	0.09	0.52	0.00	0.09				
clpP	559	Target seq	U	U	U	U	U	A	U	G	U	C	A	G	C	A	A	C	A	G	A	A	G	C	C	C	A	A				
		score																	0.73	0.16	0.64	0.15	0.93	0.31	0.78	0.25	0.70	0.91	8.02E-04	1	7.75E-05	1
rpoA	200	Target seq	A	G	A	A	G	G	A	A	C	A	U	G	U	A	U	U	A	C	A	C	G	U	G	C	A	A				
		score																	0.73	0.31	0.64	0.23	0.93	0.45	0.14	0.25	0.70	0.91	4.37E-03	3	1.80E-04	2
**Gene name**	CRR21	motifs							P	L	S	P	L	S	P	L	S	P	L	S	P	P	P	L	S	P	L2	S2				
**AGI number**	At5g55740	6							F	G	T	A	P	S	N	S	T	N	A	S	N	N	T	T	T	N	T	G				
**Type**	E	1'							G	N	N	D	C	N	T	D	D	D	D	D	N	N	N	L	E	D	C	D				
**Species**	At	A							0.25	0.34	0.64	0.25	0.04	0.70	0.04	0.25	0.09	0.09	0.25	0.06	0.08	0.08	0.73	0.40	0.45	0.09	0.70	0.09				
**Localization**	cp	C							0.25	0.14	0.06	0.25	0.06	0.14	0.69	0.25	0.07	0.25	0.25	0.00	0.55	0.55	0.05	0.19	0.19	0.25	0.16	0.07				
**ref.**	2	G							0.25	0.12	0.15	0.25	0.17	0.04	0.00	0.25	0.74	0.14	0.25	0.85	0.08	0.08	0.22	0.28	0.17	0.14	0.14	0.70				
		U							0.25	0.40	0.16	0.25	0.73	0.13	0.27	0.25	0.10	0.52	0.25	0.10	0.28	0.28	0.00	0.14	0.18	0.52	0.00	0.14				
ndhD	383	Target seq	G	U	U	A	G	C	A	A	U	G	U	A	C	A	G	C	G	G	U	C	A	A	A	U	A	G				
		score							0.25	0.34	0.16	0.25	0.73	0.70	0.69	0.25	0.74	0.25	0.25	0.85	0.28	0.55	0.73	0.40	0.45	0.52	0.70	0.70	2.82E-05	1	1.91E-07	1
**Gene name**	CRR22	motifs											S	P	L	S	P	L	S	P	L	S	P	L	S	P	L2	S2				
**AGI number**	At1g11290	6											T	H	T	T	N	V	T	N	M	N	N	V	T	N	L	G				
**Type**	DYW	1'											L	V	D	D	S	L	N	T	N	T	D	N	H	N	S	A				
**Species**	At	A											0.45	0.25	0.40	0.09	0.05	0.25	0.64	0.04	0.11	0.00	0.09	0.34	0.45	0.08	0.20	0.25				
**Localization**	cp	C											0.19	0.25	0.19	0.07	0.87	0.25	0.06	0.69	0.17	0.78	0.25	0.14	0.19	0.55	0.38	0.25				
**ref.**	3	G											0.17	0.25	0.28	0.74	0.00	0.25	0.15	0.00	0.22	0.14	0.14	0.12	0.17	0.08	0.06	0.25				
		U											0.18	0.25	0.14	0.10	0.08	0.25	0.16	0.27	0.51	0.09	0.52	0.40	0.18	0.28	0.36	0.25				
rpoB	551	Target seq	G	A	A	A	C	A	A	A	A	A	A	U	A	U	C	U	A	U	U	C	U	A	G	U	U	C				
		score											0.45	0.25	0.40	0.10	0.87	0.25	0.64	0.27	0.51	0.78	0.52	0.34	0.17	0.28	0.36	0.25	2.28E-03	3	3.64E-04	2
ndhD	887	Target seq	C	C	A	A	A	U	A	A	U	U	U	A	U	G	C	A	G	C	U	U	U	A	A	C	A	U				
		score											0.18	0.25	0.14	0.74	0.87	0.25	0.15	0.69	0.51	0.09	0.52	0.34	0.45	0.55	0.20	0.25	1.41E-03	2	1.06E-03	3
ndhB	746	Target seq	A	A	U	U	G	G	G	U	U	C	A	A	G	C	U	U	U	C	C	C	U	A	G	C	C	C				
		score											0.45	0.25	0.28	0.07	0.08	0.25	0.16	0.69	0.17	0.78	0.52	0.34	0.17	0.55	0.38	0.25	5.58E-03	4	3.40E-03	4
**Gene name**	CRR28	motifs																	S	P	L	S	P	L	S	P	L2	S2				
**AGI number**	At1g59720	6																	G	N	P	N	N	Q	N	N	V	G				
**Type**	DYW	1'																	S	D	D	S	D	D	D	N	A	D				
**Species**	At	A																	0.25	0.09	0.04	0.13	0.09	0.25	0.15	0.08	0.27	0.09				
**Localization**	cp	C																	0.25	0.25	0.06	0.50	0.25	0.25	0.23	0.55	0.21	0.07				
**ref.**	3	G																	0.25	0.14	0.17	0.09	0.14	0.25	0.07	0.08	0.09	0.70				
		U																	0.25	0.52	0.73	0.28	0.52	0.25	0.55	0.28	0.42	0.14				
ndhB	467	Target seq	C	G	G	U	A	C	A	A	U	C	C	A	A	A	U	A	A	U	U	U	A	U	G	C	A	G				
		score																	0.25	0.52	0.06	0.28	0.52	0.25	0.07	0.28	0.27	0.70	9.10E-02	11	1.16E-02	18
ndhB	467 (-5)	Target seq		C	G	G	U	A	C	A	A	U	C	C	A	A	A	U	A	A	U	U	U	A	U	G	C	A				
		score																	0.25	0.09	0.73	0.50	0.52	0.25	0.55	0.08	0.42	0.09	4.98E-02	9	2.02E-02	3
ndhD	878	Target seq	U	A	A	C	G	A	U	U	U	A	A	U	A	A	C	U	A	U	C	U	U	U	G	U	A	G				
		score																	0.25	0.52	0.73	0.28	0.09	0.25	0.07	0.55	0.27	0.70	3.82E-02	24	3.02E-02	8
ndhD	878 (-5)	Target seq		U	A	A	C	G	A	U	U	U	A	A	U	A	A	C	U	A	U	C	U	U	U	G	U	A				
		score																	0.25	0.09	0.73	0.28	0.52	0.25	0.55	0.08	0.21	0.09	8.85E-02	10	5.67E-02	10
**Gene name**	CRR4	motifs												L	P	P	L	S	S	S	S	S	P	L	S	P	L2	S2				
**AGI number**	At2g45350	6												S	T	N	S	N	N	N	N	A	N	V	V	N	V	G				
**Type**	E	1'												N	D	D	D	D	N	D	D	D	D	G	S	D	R	N				
**Species**	At	A												0.34	0.04	0.09	0.25	0.15	0.14	0.15	0.15	0.08	0.09	0.25	0.25	0.09	0.27	0.91				
**Localization**	cp	C												0.14	0.00	0.25	0.25	0.23	0.29	0.23	0.23	0.51	0.25	0.25	0.25	0.25	0.21	0.00				
**ref.**	4	G												0.12	0.93	0.14	0.25	0.07	0.41	0.07	0.07	0.34	0.14	0.25	0.25	0.14	0.09	0.00				
		U												0.40	0.03	0.52	0.25	0.55	0.16	0.55	0.55	0.07	0.52	0.25	0.25	0.52	0.42	0.09				
ndhD	2	Target seq	U	U	U	G	G	A	C	C	U	G	G	U	G	U	A	U	C	U	U	G	U	C	U	U	U	A				
		score												0.40	0.93	0.52	0.25	0.55	0.29	0.55	0.55	0.34	0.52	0.25	0.25	0.52	0.42	0.91	1.30E-05	1	2.73E-07	1
**Gene name**	LPA66	motifs																P	P	L	S	S	P	L	S	P	L2	S2				
**AGI number**	At5g48910	6																A	N	P	S	N	N	V	S	S	I	G				
**Type**	DYW	1'																N	N	D	E	S	N	D	N	S	R	D				
**Species**	At	A																0.17	0.08	0.04	0.25	0.13	0.08	0.25	0.70	0.25	0.00	0.09				
**Localization**	cp	C																0.44	0.55	0.06	0.25	0.50	0.55	0.25	0.14	0.25	0.47	0.07				
**ref.**	5	G																0.00	0.08	0.17	0.25	0.09	0.08	0.25	0.04	0.25	0.21	0.70				
		U																0.39	0.28	0.73	0.25	0.28	0.28	0.25	0.13	0.25	0.33	0.14				
PSBF	65	Target seq	U	G	U	U	C	A	U	G	G	A	C	U	A	G	C	U	G	U	A	C	C	U	A	C	C	G				
		score																0.39	0.08	0.73	0.25	0.50	0.55	0.25	0.70	0.25	0.47	0.70	6.52E-03	1	2.43E-04	1
**Gene name**	OTP80	motifs																P	L	S	S	S	P	L	S	P	L2	S2				
**AGI number**	At5g59200	6																F	T	T	L	T	T	V	N	N	V	G				
**Type**	E	1'																N	D	S	D	D	N	S	D	N	Q	D				
**Species**	At	A																0.17	0.40	0.45	0.08	0.09	0.73	0.25	0.15	0.08	0.27	0.09				
**Localization**	cp	C																0.44	0.19	0.19	0.51	0.07	0.05	0.25	0.23	0.55	0.21	0.07				
**ref.**	6	G																0.00	0.28	0.17	0.34	0.74	0.22	0.25	0.07	0.08	0.09	0.70				
		U																0.39	0.14	0.18	0.07	0.10	0.00	0.25	0.55	0.28	0.42	0.14				
rpl23	89	Target seq	U	C	A	A	U	A	U	A	C	U	U	U	U	A	A	U	G	U	C	G	A	A	U	C	A	G				
		score																0.39	0.28	0.18	0.51	0.74	0.73	0.25	0.55	0.55	0.27	0.70	2.87E-04	1	5.73E-05	1
**Gene name**	OTP81	motifs													S	P	L	S	P	L	S	S	P	L	S	P	L2	S2				
**AGI number**	At2g29760	6													S	N	P	N	N	V	N	T	N	V	S	S	T	A				
**Type**	DYW	1'													N	N	D	D	S	N	D	D	N	N	D	N	E	S				
**Species**	At	A													0.70	0.08	0.04	0.15	0.05	0.34	0.15	0.09	0.08	0.34	0.06	0.73	0.70	0.25				
**Localization**	cp	C													0.14	0.55	0.06	0.23	0.87	0.14	0.23	0.07	0.55	0.14	0.00	0.13	0.16	0.25				
**ref.**	6	G													0.04	0.08	0.17	0.07	0.00	0.12	0.07	0.74	0.08	0.12	0.85	0.00	0.14	0.25				
		U													0.13	0.28	0.73	0.55	0.08	0.40	0.55	0.10	0.28	0.40	0.10	0.14	0.00	0.25				
rps12	69553	Target seq	A	C	A	U	A	A	A	A	A	G	G	A	A	A	U	U	C	U	U	U	U	U	G	A	A	A				
		score													0.70	0.08	0.73	0.55	0.87	0.40	0.55	0.10	0.28	0.40	0.85	0.73	0.70	0.25	1.25E-03	2	5.86E-05	1
**Gene name**	OTP82	motifs														P	L	S	S	P	L	S	P	L	S	P	L2	S2				
**AGI number**	At1g08070	6														N	P	T	T	N	V	N	N	L	T	N	V	G				
**Type**	DYW	1'														N	D	D	D	D	N	D	N	A	S	D	K	D				
**Species**	At	A														0.08	0.04	0.09	0.09	0.09	0.34	0.15	0.08	0.20	0.45	0.09	0.27	0.09				
**Localization**	cp	C														0.55	0.06	0.07	0.07	0.25	0.14	0.23	0.55	0.32	0.19	0.25	0.21	0.07				
**ref.**	6	G														0.08	0.17	0.74	0.74	0.14	0.12	0.07	0.08	0.00	0.17	0.14	0.09	0.70				
		U														0.28	0.73	0.10	0.10	0.52	0.40	0.55	0.28	0.48	0.18	0.52	0.42	0.14				
ndhB	836	Target seq	U	G	U	U	A	C	U	U	C	G	A	A	A	G	U	A	G	C	U	G	C	U	U	U	A	G				
		score														0.08	0.73	0.09	0.74	0.25	0.40	0.07	0.55	0.48	0.18	0.52	0.27	0.70	5.85E-02	9	3.43E-03	4
ndhG	50	Target seq	A	C	A	U	G	A	U	U	U	U	C	U	U	U	U	A	G	U	U	U	U	U	C	U	G	G				
		score														0.28	0.73	0.09	0.74	0.52	0.40	0.55	0.28	0.48	0.19	0.52	0.09	0.70	6.40E-03	1	5.42E-04	1

		PPR position	26	25	24	23	22	21	20	19	18	17	16	15	14	13	12	11	10	9	8	7	6	5	4	3	2	1	P-value	Rank without L, L2 and S2	P-value	Rank with L, L2 and S2
**Gene name**	OTP84	motifs									P	L	S	P	L	S	P	L	S	P	L	S	P	L	S	P	L2	S2				
**AGI number**	At3g57430	6									I	P	N	N	V	N	N	S	S	N	A	N	N	M	S	N	I	A				
**Type**	DYW	1'									D	S	N	S	N	D	D	N	K	N	D	D	N	D	N	N	S	N				
**Species**	At	A									0.25	0.04	0.14	0.05	0.34	0.15	0.09	0.34	0.25	0.08	0.25	0.15	0.08	0.11	0.70	0.08	0.00	0.91				
**Localization**	cp	C									0.25	0.06	0.29	0.87	0.14	0.23	0.25	0.14	0.25	0.55	0.25	0.23	0.55	0.17	0.14	0.55	0.47	0.00				
**ref.**	6	G									0.25	0.17	0.41	0.00	0.12	0.07	0.14	0.12	0.25	0.08	0.25	0.07	0.08	0.22	0.04	0.08	0.21	0.00				
		U									0.25	0.73	0.16	0.08	0.40	0.55	0.52	0.40	0.25	0.28	0.25	0.55	0.28	0.51	0.13	0.28	0.33	0.09				
ndhB	1481	Target seq	G	A	U	U	G	U	A	U	G	U	G	U	G	A	U	A	G	C	A	U	C	U	A	C	U	A				
		score									0.25	0.73	0.41	0.08	0.12	0.15	0.52	0.34	0.25	0.55	0.25	0.55	0.55	0.51	0.70	0.55	0.33	0.91	1.31E-03	3	5.29E-05	2
ndhF	290	Target seq	C	U	U	U	A	U	C	G	A	U	C	C	A	C	U	U	A	C	U	U	C	U	A	U	U	A				
		score									0.25	0.73	0.29	0.87	0.34	0.23	0.52	0.40	0.25	0.55	0.25	0.55	0.55	0.51	0.70	0.28	0.33	0.91	2.09E-04	1	9.91E-07	1
psbZ	50	Target seq	G	G	C	A	G	U	U	U	U	U	G	C	A	U	U	A	A	U	U	A	U	U	A	C	U	U				
		score									0.25	0.73	0.41	0.87	0.34	0.55	0.52	0.34	0.25	0.28	0.25	0.15	0.28	0.51	0.70	0.55	0.33	0.09	9.56E-04	2	1.45E-04	3
**Gene name**	OTP85	motifs																	S	P	L	S	P	L	S	P	L2	S2				
**AGI number**	At2g02980	6																	A	N	P	P	N	L	T	S	L	G				
**Type**	DYW	1'																	D	D	N	C	N	Y	D	D	S	T				
**Species**	At	A																	0.08	0.09	0.04	0.25	0.08	0.20	0.09	0.11	0.20	0.23				
**Localization**	cp	C																	0.51	0.25	0.06	0.25	0.55	0.32	0.07	0.34	0.38	0.18				
**ref.**	6	G																	0.34	0.14	0.17	0.25	0.08	0.00	0.74	0.45	0.06	0.00				
		U																	0.07	0.52	0.73	0.25	0.28	0.48	0.10	0.10	0.36	0.60				
ndhD	674	Target seq	U	G	G	A	U	U	U	C	U	U	A	U	U	G	C	U	U	U	U	G	C	C	G	U	C	A				
		score																	0.07	0.52	0.73	0.25	0.55	0.32	0.74	0.10	0.38	0.23	3.12E-02	6	6.14E-03	3
**Gene name**	OTP86	motifs							P	S	P	L	S	P	L	S	P	L	S	P	L	S	P	L	S	P	L2	S2				
**AGI number**	At3g63370	6							A	G	N	P	N	N	V	N	N	T	N	T	G	N	T	L	V	T	L	V				
**Type**	DYW	1'							L	T	G	T	D	N	E	D	D	N	D	D	D	D	D	E	G	D	W	T				
**Species**	At	A							0.25	0.25	0.25	0.04	0.15	0.08	0.25	0.15	0.09	0.40	0.15	0.04	0.25	0.15	0.04	0.20	0.25	0.04	0.20	0.23				
**Localization**	cp	C							0.25	0.25	0.25	0.06	0.23	0.55	0.25	0.23	0.25	0.19	0.23	0.00	0.25	0.23	0.00	0.32	0.25	0.00	0.38	0.18				
**ref.**	6	G							0.25	0.25	0.25	0.17	0.07	0.08	0.25	0.07	0.14	0.28	0.07	0.93	0.25	0.07	0.93	0.00	0.25	0.93	0.06	0.00				
		U							0.25	0.25	0.25	0.73	0.55	0.28	0.25	0.55	0.52	0.14	0.55	0.03	0.25	0.55	0.03	0.48	0.25	0.03	0.36	0.60				
rps14	80	Target seq	A	A	A	A	A	A	A	U	A	U	C	A	U	U	U	G	A	U	U	C	G	U	C	G	A	U				
		score							0.25	0.25	0.25	0.73	0.23	0.08	0.25	0.55	0.52	0.28	0.15	0.03	0.25	0.23	0.93	0.48	0.25	0.93	0.20	0.60	9.20E-03	2	1.35E-03	1
**Gene name**	RARE1	motifs												P	L	S	P	L	S	P	L	S	P	L	S	P	L2	S2				
**AGI number**	At5g13270	6												N	Q	N	T	T	T	T	S	T	S	T	S	T	I	D				
**Type**	DYW	1'												S	S	N	P	N	K	D	E	N	N	S	D	N	T	D				
**Species**	At	A												0.05	0.25	0.14	0.35	0.40	0.45	0.04	0.25	0.64	0.73	0.40	0.06	0.73	0.00	0.09				
**Localization**	cp	C												0.87	0.25	0.29	0.21	0.19	0.19	0.00	0.25	0.06	0.13	0.19	0.00	0.05	0.47	0.07				
**ref.**	7	G												0.00	0.25	0.41	0.22	0.28	0.17	0.93	0.25	0.15	0.00	0.28	0.85	0.22	0.21	0.70				
		U												0.08	0.25	0.16	0.22	0.14	0.18	0.03	0.25	0.16	0.14	0.14	0.10	0.00	0.33	0.14				
accD	794	Target seq	A	A	U	G	A	G	U	A	G	U	U	C	A	G	A	A	A	G	A	A	U	C	G	A	G	C				
		score												0.87	0.25	0.41	0.35	0.40	0.45	0.93	0.25	0.64	0.14	0.19	0.85	0.73	0.21	0.07	6.03E-05	1	2.50E-04	1
**Gene name**	YS1	motifs											P	L	S	P	L	S	P	L	S	S	P	L	S	P	L2	S2				
**AGI number**	At3g22690	6											N	P	N	T	V	S	N	L	D	N	N	M	T	T	V	G				
**Type**	DYW	1'											D	D	N	N	N	N	D	G	T	N	D	D	D	D	E	N				
**Species**	At	A											0.09	0.04	0.14	0.73	0.34	0.70	0.09	0.20	0.25	0.14	0.09	0.11	0.09	0.04	0.27	0.91				
**Localization**	cp	C											0.25	0.06	0.29	0.05	0.14	0.14	0.25	0.32	0.25	0.29	0.25	0.17	0.07	0.00	0.21	0.00				
**ref.**	8	G											0.14	0.17	0.41	0.22	0.12	0.04	0.14	0.00	0.25	0.41	0.14	0.22	0.74	0.93	0.09	0.00				
		U											0.52	0.73	0.16	0.00	0.40	0.13	0.52	0.48	0.25	0.16	0.52	0.51	0.10	0.03	0.42	0.09				
rpoB	338	Target seq	G	U	U	U	G	A	C	A	U	G	G	U	U	U	A	C	G	U	G	U	U	A	C	U	G	G				
		score											0.09	0.73	0.41	0.73	0.34	0.13	0.52	0.32	0.25	0.29	0.52	0.51	0.74	0.93	0.27	0.91	1.03E-03	1	1.63E-05	1
**Gene name**	AHG11	motifs														S	P	L	S	S	S	S	P	L	S	P	L2	S2				
**AGI number**	AT2g44880	6														T	N	T	T	T	N	T	N	L	T	N	L	G				
**Type**	E	1'														D	D	D	S	D	T	N	D	K	Q	D	K	N				
**Species**	At	A														0.09	0.09	0.40	0.45	0.09	0.00	0.64	0.09	0.20	0.45	0.09	0.20	0.91				
**Localization**	mt	C														0.07	0.25	0.19	0.19	0.07	0.78	0.06	0.25	0.32	0.19	0.25	0.38	0.00				
**ref.**	9	G														0.74	0.14	0.28	0.17	0.74	0.14	0.15	0.14	0.00	0.17	0.14	0.06	0.00				
		U														0.10	0.52	0.14	0.18	0.10	0.09	0.16	0.52	0.48	0.18	0.52	0.36	0.09				
nad4	376	Target seq	G	G	A	A	A	G	A	G	U	A	U	A	U	U	A	U	A	G	C	A	U	U	U	U	U	A				
		score														0.10	0.09	0.14	0.45	0.74	0.78	0.64	0.52	0.48	0.18	0.52	0.36	0.91	5.39E-03	7	8.90E-04	3
**Gene name**	MEF1	motifs																L	S	P	L	S	P	L	S	P	L2	S2				
**AGI number**	At5g52630	6																C	N	S	P	S	S	S	S	N	L	A				
**Type**	DYW	1'																I	S	D	D	N	N	S	N	N	T	T				
**Species**	At	A																0.25	0.13	0.11	0.04	0.70	0.73	0.25	0.70	0.08	0.20	0.23				
**Localization**	mt	C																0.25	0.50	0.34	0.06	0.14	0.13	0.25	0.14	0.55	0.38	0.18				
**ref.**	10	G																0.25	0.09	0.45	0.17	0.04	0.00	0.25	0.04	0.08	0.06	0.00				
		U																0.25	0.28	0.10	0.73	0.13	0.14	0.25	0.13	0.28	0.36	0.60				
nad2	1160	Target seq	G	G	G	C	G	C	U	C	U	A	G	C	C	A	A	A	A	C	G	A	A	U	C	C	U	A				
		score																0.25	0.13	0.34	0.17	0.70	0.73	0.25	0.14	0.55	0.36	0.23	1.00E-02	17	8.43E-03	19
nad7	963	Target seq	C	A	U	U	U	C	G	A	A	C	U	U	U	A	U	A	C	A	G	A	A	G	G	U	U	U				
		score																0.25	0.50	0.11	0.17	0.70	0.73	0.25	0.04	0.28	0.36	0.60	3.47E-02	53	1.36E-02	33
rps4	956	Target seq	C	A	A	A	A	G	G	A	U	C	G	A	A	C	U	A	C	C	U	A	C	U	C	A	U	U				
		score																0.25	0.50	0.34	0.73	0.70	0.13	0.25	0.14	0.08	0.36	0.60	4.96E-02	76	7.21E-03	13
**Gene name**	MEF3	motifs														S	P	L	S	P	L	S	P	L	S	P	L2	S2				
**AGI number**	At1g06140	6														S	N	V	P	G	I	A	T	A	T	S	V	A				
**Type**	E	1'														N	D	D	N	D	S	N	N	D	N	N	E	M				
**Species**	At	A														0.70	0.09	0.25	0.53	0.25	0.08	0.53	0.73	0.25	0.64	0.73	0.27	0.25				
**Localization**	mt	C														0.14	0.25	0.25	0.00	0.25	0.31	0.00	0.05	0.25	0.06	0.13	0.21	0.25				
**ref.**	11	G														0.04	0.14	0.25	0.00	0.25	0.16	0.00	0.22	0.25	0.15	0.00	0.09	0.25				
		U														0.13	0.52	0.25	0.47	0.25	0.45	0.47	0.00	0.25	0.16	0.14	0.42	0.25				
atp4	89	Target seq	U	A	U	U	U	G	U	G	C	A	U	U	A	A	G	U	U	C	G	A	A	G	A	A	G	A				
		score														0.70	0.14	0.25	0.47	0.25	0.16	0.53	0.73	0.25	0.64	0.73	0.09	0.25	3.16E-04	1	1.93E-03	3

		PPR position	26	25	24	23	22	21	20	19	18	17	16	15	14	13	12	11	10	9	8	7	6	5	4	3	2	1	P-value	Rank without L, L2 and S2	P-value	Rank with L, L2 and S2
**Gene name**	MEF7	motifs					S	P	L	S	P	L	S	P	L	S	P	L	S	P	L	S	P	L	S	P	L2	S2				
**AGI number**	At5g09950	6					N	A	V	N	N	G	S	N	L	N	N	I	N	N	S	N	N	A	S	N	V	S				
**Type**	DYW	1'					N	N	D	N	T	D	N	S	M	D	G	N	D	N	E	D	D	D	N	D	R	N				
**Species**	At	A					0.14	0.17	0.25	0.14	0.04	0.25	0.70	0.05	0.20	0.15	0.25	0.08	0.15	0.08	0.25	0.15	0.09	0.25	0.70	0.09	0.27	0.91				
**Localization**	mt	C					0.29	0.44	0.25	0.29	0.69	0.25	0.14	0.87	0.32	0.23	0.25	0.31	0.23	0.55	0.25	0.23	0.25	0.25	0.14	0.25	0.21	0.00				
**ref.**	12	G					0.41	0.00	0.25	0.41	0.00	0.25	0.04	0.00	0.00	0.07	0.25	0.16	0.07	0.08	0.25	0.07	0.14	0.25	0.04	0.14	0.09	0.00				
		U					0.16	0.39	0.25	0.16	0.27	0.25	0.13	0.08	0.48	0.55	0.25	0.45	0.55	0.28	0.25	0.55	0.52	0.25	0.13	0.52	0.42	0.09				
ccmB	28	Target seq	A	A	A	U	G	A	G	A	C	G	A	C	U	U	U	U	U	U	U	U	G	A	A	C	U	A				
		score					0.41	0.17	0.25	0.14	0.69	0.25	0.70	0.87	0.48	0.55	0.25	0.45	0.55	0.28	0.25	0.55	0.14	0.25	0.70	0.25	0.42	0.91	2.34E-04	2	8.03E-06	2
cob	325	Target seq	U	U	U	A	C	C	U	U	C	A	U	A	U	U	U	U	U	C	G	U	G	G	U	C	U	A				
		score					0.29	0.44	0.25	0.16	0.69	0.25	0.13	0.05	0.48	0.55	0.25	0.45	0.55	0.55	0.25	0.55	0.14	0.25	0.13	0.25	0.42	0.91	9.34E-03	32	7.56E-04	10
nad2	1433	Target seq	A	C	U	A	G	C	A	A	U	G	A	C	U	U	C	C	U	U	U	U	U	C	A	U	U	A				
		score					0.41	0.44	0.25	0.14	0.27	0.25	0.70	0.87	0.48	0.55	0.25	0.31	0.55	0.28	0.25	0.55	0.52	0.25	0.70	0.52	0.42	0.91	1.10E-05	1	4.06E-07	1
nad4L	41	Target seq	C	A	A	A	U	A	U	U	U	C	A	C	A	U	U	U	U	C	U	A	U	G	A	U	U	A				
		score					0.16	0.17	0.25	0.16	0.27	0.25	0.70	0.87	0.20	0.55	0.25	0.45	0.55	0.55	0.25	0.15	0.52	0.25	0.70	0.52	0.42	0.91	4.65E-04	3	4.94E-05	3

		PPR position	26	25	24	23	22	21	20	19	18	17	16	15	14	13	12	11	10	9	8	7	6	5	4	3	2	1	P-value	Rank without L, L2 and S2	P-value	Rank with L, L2 and S2
Gene name	MEF8	motifs																						L	S	P	L2	S2				
AGI number	At2g25580	6																						L	H	C	R	V				
Type	DYW	1'																						D	N	D	S	N				
Species	At	A																						0.20	0.53	0.25	0.25	0.91				
Localization	mt	C																						0.32	0.00	0.25	0.25	0.00				
ref.	13	G																						0.00	0.00	0.25	0.25	0.00				
		U																						0.48	0.47	0.25	0.25	0.09				
nad5	676	Target seq	A	U	G	C	C	A	U	A	A	G	U	C	U	U	A	U	U	U	G	U	A	U	U	U	U	A				
		score																						0.48	0.47	0.25	0.25	0.91	1.13E-01	213 (16)	1.13E-02	23 (5)
nad6	95	Target seq	U	C	C	G	G	U	A	C	A	U	U	C	C	G	U	U	U	U	G	U	U	U	U	U	C	A				
		score																						0.48	0.47	0.25	0.25	0.91	>1	P>1	3.39E-03	6 (2)
Gene name	MEF9	motifs												S	P	S	S	L	S	S	S	S	P	L	S	P	L2	S2				
AGI number	At1g62260	6												N	N	N	S	A	N	N	N	N	N	T	N	N	V	S				
Type	E	1'												D	D	N	L	L	N	D	D	H	D	D	E	S	Q	D				
Species	At	A												0.15	0.09	0.14	0.25	0.25	0.14	0.15	0.15	0.25	0.09	0.40	0.25	0.05	0.27	0.09				
Localization	mt	C												0.23	0.25	0.29	0.25	0.25	0.29	0.23	0.23	0.25	0.25	0.19	0.25	0.87	0.21	0.07				
ref.	14	G												0.07	0.14	0.41	0.25	0.25	0.41	0.07	0.07	0.25	0.14	0.28	0.25	0.00	0.09	0.70				
		U												0.55	0.52	0.16	0.25	0.25	0.16	0.55	0.55	0.25	0.52	0.14	0.25	0.08	0.42	0.14				
nad7	200	Target seq	C	A	A	A	A	C	U	U	A	U	C	U	U	C	A	A	G	C	U	U	U	A	C	C	U	U				
		score												0.55	0.52	0.29	0.25	0.25	0.41	0.23	0.55	0.25	0.52	0.40	0.25	0.87	0.42	0.14	2.41E-04	5	2.05E-04	1
Gene name	MEF10	motifs															P	L	S	P	L	S	P	L	S	P	L2	S2				
AGI number	At3g11460	6															N	P	T	N	L	N	N	V	N	T	V	S				
Type	DYW	1'															D	E	L	D	E	G	D	N	S	D	G	D				
Species	At	A															0.09	0.04	0.45	0.09	0.20	0.25	0.09	0.34	0.13	0.04	0.27	0.09				
Localization	mt	C															0.25	0.06	0.19	0.25	0.32	0.25	0.25	0.14	0.50	0.00	0.21	0.07				
ref.	15	G															0.14	0.17	0.17	0.14	0.00	0.25	0.14	0.12	0.09	0.93	0.09	0.70				
		U															0.52	0.73	0.18	0.52	0.48	0.25	0.52	0.40	0.28	0.03	0.42	0.14				
nad2	842	Target seq	C	U	C	U	A	U	U	U	U	U	G	C	U	A	A	U	A	U	U	U	U	A	C	G	U	G				
		score															0.09	0.73	0.45	0.52	0.48	0.25	0.52	0.34	0.50	0.93	0.42	0.70	5.08E-03	9	4.82E-05	2
Gene name	MEF11	motifs													L	S	P	L	S	P	L	S	P	L	S	P	L2	S2				
AGI number	At4g14850	6													G	N	T	P	C	N	C	N	C	S	S	N	V	S				
Type	DYW	1'													P	N	N	D	N	N	D	N	S	T	N	N	G	T				
Species	At	A													0.25	0.14	0.73	0.04	0.53	0.08	0.25	0.14	0.25	0.25	0.70	0.08	0.27	0.23				
Localization	mt	C													0.25	0.29	0.05	0.06	0.00	0.55	0.25	0.29	0.25	0.25	0.14	0.55	0.21	0.18				
ref.	16	G													0.25	0.41	0.22	0.17	0.00	0.08	0.25	0.41	0.25	0.25	0.04	0.08	0.09	0.00				
		U													0.25	0.16	0.00	0.73	0.47	0.28	0.25	0.16	0.25	0.25	0.13	0.28	0.42	0.60				
ccmFN2	344	Target seq	G	A	C	C	U	U	G	U	U	U	C	U	U	A	A	U	A	U	U	G	U	G	A	C	U	U				
		score													0.25	0.14	0.73	0.73	0.53	0.28	0.25	0.41	0.25	0.25	0.70	0.55	0.42	0.60	1.81E-03	2	7.69E-05	1
ccmC	568	Target seq	U	U	A	G	C	U	G	A	U	C	U	G	G	U	A	C	A	U	C	A	A	U	A	C	A	U				
		score													0.25	0.16	0.73	0.06	0.53	0.28	0.25	0.14	0.25	0.25	0.70	0.55	0.27	0.60	5.37E-03	14	6.01E-03	17
cox3	422	Target seq	A	A	U	C	C	C	U	U	U	U	C	U	U	A	A	U	A	C	C	C	U	U	A	U	U	C				
		score													0.25	0.14	0.73	0.73	0.53	0.55	0.25	0.29	0.25	0.25	0.70	0.28	0.42	0.18	1.98E-03	3	5.06E-04	3
matR	1730	Target seq	C	G	U	C	A	G	C	G	A	C	G	A	A	G	A	C	A	U	C	G	U	A	A	A	U	U				
		score													0.25	0.41	0.73	0.06	0.53	0.28	0.25	0.41	0.25	0.25	0.70	0.08	0.42	0.60	3.98E-03	8	3.48E-03	10
nad4	124	Target seq	C	A	A	G	A	A	U	A	C	G	A	C	U	G	A	U	A	C	G	A	U	U	A	A	U	U				
		score													0.25	0.41	0.73	0.73	0.53	0.55	0.25	0.14	0.25	0.25	0.70	0.08	0.42	0.60	6.18E-03	15	3.88E-04	2
Gene name	MEF14	motifs															P	L	S	P	L	S	P	L	S	P	L2	S2				
AGI number	At3g26780	6															N	P	S	T	V	N	N	S	T	T	V	G				
Type	DYW	1'															T	D	N	D	G	D	N	D	N	N	G	D				
Species	At	A															0.04	0.04	0.70	0.04	0.25	0.15	0.08	0.25	0.64	0.73	0.27	0.09				
Localization	mt	C															0.69	0.06	0.14	0.00	0.25	0.23	0.55	0.25	0.06	0.05	0.21	0.07				
ref.	17	G															0.00	0.17	0.04	0.93	0.25	0.07	0.08	0.25	0.15	0.22	0.09	0.70				
		U															0.27	0.73	0.13	0.03	0.25	0.55	0.28	0.25	0.16	0.00	0.42	0.14				
matR	1895	Target seq	G	A	A	U	A	U	A	A	U	C	C	U	C	A	A	G	U	A	C	U	C	C	A	A	A	G				
		score															0.04	0.17	0.13	0.04	0.25	0.55	0.55	0.25	0.64	0.73	0.27	0.70	2.46E-02	38	1.22E-02	29
Gene name	MEF18	motifs										L	S	S	P	L	S	S	P	L	S	S	P	L	S	P	L2	S2				
AGI number	At5g19020	6										V	N	N	T	A	T	N	G	V	A	N	N	V	A	N	V	G				
Type	E	1'										N	D	S	N	R	N	D	S	Y	H	D	D	N	T	N	D	D				
Species	At	A										0.34	0.15	0.13	0.73	0.25	0.64	0.15	0.25	0.25	0.25	0.15	0.09	0.34	0.25	0.08	0.27	0.09				
Localization	mt	C										0.14	0.23	0.50	0.05	0.25	0.06	0.23	0.25	0.25	0.25	0.23	0.25	0.14	0.25	0.55	0.21	0.07				
ref.	18	G										0.12	0.07	0.09	0.22	0.25	0.15	0.07	0.25	0.25	0.25	0.07	0.14	0.12	0.25	0.08	0.09	0.70				
		U										0.40	0.55	0.28	0.00	0.25	0.16	0.55	0.25	0.25	0.25	0.55	0.52	0.40	0.25	0.28	0.42	0.14				
nad4	1355	Target seq	A	C	C	U	G	A	U	U	U	C	C	U	C	C	A	U	A	A	A	U	U	C	U	C	C	G				
		score										0.14	0.23	0.28	0.05	0.25	0.64	0.55	0.25	0.25	0.25	0.55	0.52	0.14	0.25	0.55	0.21	0.70	3.89E-03	9	5.13E-03	12
Gene name	MEF19	motifs														S	P	L	S	P	L	S	P	L	S	P	L2	S2				
AGI number	At3g05240	6														S	N	P	T	G	V	T	N	L	C	T	L	G				
Type	E	1'														S	D	N	N	N	N	T	D	D	D	D	T	N				
Species	At	A														0.25	0.09	0.04	0.64	0.17	0.34	0.45	0.09	0.20	0.08	0.04	0.20	0.91				
Localization	mt	C														0.25	0.25	0.06	0.06	0.44	0.14	0.19	0.25	0.32	0.51	0.00	0.38	0.00				
ref.	18	G														0.25	0.14	0.17	0.15	0.00	0.12	0.17	0.14	0.00	0.34	0.93	0.06	0.00				
		U														0.25	0.52	0.73	0.16	0.39	0.40	0.18	0.52	0.48	0.07	0.03	0.36	0.09				
ccmB	566	Target seq	G	U	U	U	C	A	U	G	U	U	C	U	U	U	U	A	U	U	G	A	U	U	G	G	U	U				
		score														0.25	0.52	0.04	0.16	0.39	0.12	0.45	0.52	0.48	0.34	0.93	0.36	0.09	3.28E-03	4	9.54E-03	34
ccmB	566 (-5)	Target seq	G	G	U	U	U	C	A	U	G	U	U	C	U	U	U	U	A	U	U	G	A	U	U	G	G	U				
		score														0.25	0.52	0.73	0.64	0.39	0.40	0.17	0.09	0.48	0.07	0.93	0.06	0.09	3.64E-02	46	1.16E-02	25
Gene name	MEF20	motifs																			L	S	P	L	S	P	L2	S2				
AGI number	At3g18970	6																			V	T	N	V	T	T	T	G				
Type	E	1'																			S	T	T	D	N	N	V	D				
Species	At	A																			0.25	0.45	0.04	0.25	0.64	0.73	0.70	0.09				
Localization	mt	C																			0.25	0.19	0.69	0.25	0.06	0.05	0.16	0.07				
ref.	18	G																			0.25	0.17	0.00	0.25	0.15	0.22	0.14	0.70				
		U																			0.25	0.18	0.27	0.25	0.16	0.00	0.00	0.14				
rps4	226	Target seq	A	U	A	U	A	C	A	A	U	U	A	C	A	A	A	C	U	A	C	A	C	G	A	A	A	G				
		score																			0.25	0.45	0.69	0.25	0.64	0.73	0.70	0.70	3.00E-04	1	1.41E-05	1
Gene name	MEF21	motifs																S	P	L	S	S	P	L	S	P	L2	S2				
AGI number	At2g20540	6																T	N	P	N	N	T	I	N	S	L	G				
Type	E	1'																N	D	H	D	T	D	Q	D	N	K	D				
Species	At	A																0.64	0.09	0.04	0.15	0.00	0.04	0.08	0.15	0.73	0.20	0.09				
Localization	mt	C																0.06	0.25	0.06	0.23	0.78	0.00	0.31	0.23	0.13	0.38	0.07				
ref.	18	G																0.15	0.14	0.17	0.07	0.14	0.93	0.16	0.07	0.00	0.06	0.70				
		U																0.16	0.52	0.73	0.55	0.09	0.03	0.45	0.55	0.14	0.36	0.14				
cox3	257	Target seq	A	G	U	C	G	U	A	C	A	A	U	U	A	G	G	A	C	U	U	C	G	A	U	A	U	G				
		score																0.64	0.25	0.73	0.55	0.78	0.93	0.08	0.55	0.73	0.36	0.70	4.99E-05	1	1.64E-05	1

		PPR position	26	25	24	23	22	21	20	19	18	17	16	15	14	13	12	11	10	9	8	7	6	5	4	3	2	1	P-value	Rank without L, L2 and S2	P-value	Rank with L, L2 and S2
**Gene name**	MEF22	motifs														S	P	L	S	P	L	S	P	L	S	P	L2	S2				
**AGI number**	At3g12770	6														T	N	P	N	T	V	I	N	T	S	S	L	A				
**Type**	DYW	1'														Q	D	D	T	D	E	N	D	D	D	N	Q	G				
**Species**	At	A														0.45	0.09	0.04	0.00	0.04	0.25	0.53	0.09	0.40	0.06	0.73	0.20	0.25				
**Localization**	mt	C														0.19	0.25	0.06	0.78	0.00	0.25	0.00	0.25	0.19	0.00	0.13	0.38	0.25				
**ref.**	18	G														0.17	0.14	0.17	0.14	0.93	0.25	0.00	0.14	0.28	0.85	0.00	0.06	0.25				
		U														0.18	0.52	0.73	0.09	0.03	0.25	0.47	0.52	0.14	0.10	0.14	0.36	0.25				
nad3	149	Target seq	G	U	C	G	G	C	C	U	A	C	G	A	A	U	G	U	G	G	U	U	U	C	G	A	U	C				
		score														0.18	0.14	0.73	0.14	0.93	0.25	0.47	0.52	0.19	0.85	0.73	0.36	0.25	1.33E-03	4	3.90E-04	2
**Gene name**	MEF29	motifs											S	P	L	S	P	L	S	P	L	S	P	L	S	P	L2	S2				
**AGI number**	At4g30700	6											T	N	A	S	N	L	T	N	V	T	N	T	T	N	L	A				
**Type**	DYW	1'											D	N	E	D	D	H	D	R	H	S	N	S	N	T	S	G				
**Species**	At	A											0.09	0.08	0.25	0.06	0.09	0.20	0.09	0.25	0.25	0.45	0.08	0.40	0.64	0.04	0.20	0.25				
**Localization**	mt	C											0.07	0.55	0.25	0.00	0.25	0.32	0.07	0.25	0.25	0.19	0.55	0.19	0.06	0.69	0.38	0.25				
**ref.**	19	G											0.74	0.08	0.25	0.85	0.14	0.00	0.74	0.25	0.25	0.17	0.08	0.28	0.15	0.00	0.06	0.25				
		U											0.10	0.28	0.25	0.10	0.52	0.48	0.10	0.25	0.25	0.18	0.28	0.14	0.16	0.27	0.36	0.25				
cob	908	Target seq	C	A	A	A	G	C	G	G	G	A	G	G	U	G	U	A	G	C	C	G	C	A	A	U	A	G				
		score											0.74	0.08	0.25	0.85	0.52	0.20	0.74	0.25	0.25	0.17	0.55	0.40	0.64	0.27	0.20	0.25	2.70E-04	1	2.54E-04	3
nad5	1550	Target seq	C	G	A	A	U	C	C	G	A	G	U	U	U	G	C	U	G	C	U	C	C	A	A	C	C	A				
		score											0.10	0.28	0.25	0.85	0.25	0.48	0.74	0.25	0.25	0.19	0.55	0.40	0.64	0.69	0.38	0.25	6.72E-04	3	1.26E-04	2
**Gene name**	OTP87	motifs					S	P	L	S	P	L	S	P	L	S	P	L	S	P	L	S	P	L	S	P	L2	S2				
**AGI number**	At1g74600	6					K	N	G	S	N	S	T	T	T	A	N	C	S	A	A	S	S	S	S	T	V	V				
**Type**	E	1'					D	N	Y	N	D	D	S	N	D	R	D	D	D	D	G	D	D	E	D	D	E	D				
**Species**	At	A					0.08	0.08	0.25	0.70	0.09	0.25	0.45	0.73	0.40	0.25	0.09	0.25	0.06	0.25	0.25	0.06	0.11	0.25	0.06	0.04	0.27	0.09				
**Localization**	mt	C					0.51	0.55	0.25	0.14	0.25	0.25	0.19	0.05	0.19	0.25	0.25	0.25	0.00	0.25	0.25	0.00	0.34	0.25	0.00	0.00	0.21	0.07				
**ref.**	20	G					0.34	0.08	0.25	0.04	0.14	0.25	0.17	0.22	0.28	0.25	0.14	0.25	0.85	0.25	0.25	0.85	0.45	0.25	0.85	0.93	0.09	0.70				
		U					0.07	0.28	0.25	0.13	0.52	0.25	0.18	0.00	0.14	0.25	0.52	0.25	0.10	0.25	0.25	0.10	0.10	0.25	0.10	0.03	0.42	0.14				
atp1	1178	Target seq	G	A	A	A	G	C	U	A	U	G	A	A	A	C	A	A	G	U	A	U	G	C	G	G	U	A				
		score					0.34	0.55	0.25	0.70	0.52	0.25	0.45	0.73	0.40	0.25	0.09	0.25	0.85	0.25	0.25	0.10	0.45	0.25	0.85	0.93	0.42	0.09	4.98E-05	1	4.59E-05	1
nad7	24	Target seq	U	U	U	C	C	C	A	U	G	A	C	G	A	C	U	A	G	G	A	A	A	A	G	G	C	A				
		score					0.51	0.55	0.25	0.13	0.14	0.25	0.19	0.22	0.40	0.25	0.52	0.25	0.85	0.25	0.25	0.06	0.11	0.25	0.85	0.93	0.21	0.09	2.23E-03	4	2.64E-03	5
**Gene name**	REME1	motifs																L	S	P	L	S	P	L	S	P	L2	S2				
**AGI number**	At2g03880	6																S	N	T	S	S	N	T	N	S	V	G				
**Type**	DYW	1'																M	N	N	D	D	E	D	D	N	V	D				
**Species**	At	A																0.25	0.14	0.73	0.25	0.06	0.25	0.40	0.15	0.73	0.27	0.09				
**Localization**	mt	C																0.25	0.29	0.05	0.25	0.00	0.25	0.19	0.23	0.13	0.21	0.07				
**ref.**	21	G																0.25	0.41	0.22	0.25	0.85	0.25	0.28	0.07	0.00	0.09	0.70				
		U																0.25	0.16	0.00	0.25	0.10	0.25	0.14	0.55	0.14	0.42	0.14				
nad2	558	Target seq	U	U	G	A	A	A	U	A	U	U	U	G	A	U	C	U	U	A	G	G	U	G	C	A	U	U				
		score																0.25	0.16	0.73	0.25	0.85	0.25	0.28	0.23	0.73	0.42	0.14	3.40E-03	7	2.73E-03	10
**Gene name**	SLG1	motifs						L	S	P	L	S	P	L	S	P	L	S	P	L	S	S	P	L	S	P	L2	S2				
**AGI number**	At5g08490	6						L	K	N	A	N	N	A	N	N	I	N	N	L	N	N	S	M	T	T	T	A				
**Type**	E	1'						C	D	S	D	D	N	H	D	D	D	D	D	E	T	D	N	I	D	D	T	N				
**Species**	At	A						0.20	0.08	0.05	0.25	0.15	0.08	0.25	0.15	0.09	0.08	0.15	0.09	0.20	0.00	0.15	0.73	0.11	0.09	0.04	0.70	0.91				
**Localization**	mt	C						0.32	0.51	0.87	0.25	0.23	0.55	0.25	0.23	0.25	0.31	0.23	0.25	0.32	0.78	0.23	0.13	0.17	0.07	0.00	0.16	0.00				
**ref.**	22	G						0.00	0.34	0.00	0.25	0.07	0.08	0.25	0.07	0.14	0.16	0.07	0.14	0.00	0.14	0.07	0.00	0.22	0.74	0.93	0.14	0.00				
		U						0.48	0.07	0.08	0.25	0.55	0.28	0.25	0.55	0.52	0.45	0.55	0.52	0.48	0.09	0.55	0.14	0.51	0.10	0.03	0.00	0.09				
nad3	250	Target seq	A	A	G	U	A	A	C	C	U	U	U	U	U	C	U	U	U	C	C	U	U	G	G	G	C	A				
		score						0.20	0.51	0.87	0.25	0.55	0.28	0.25	0.55	0.25	0.45	0.55	0.52	0.32	0.78	0.55	0.14	0.22	0.74	0.93	0.16	0.91	2.14E-06	1	8.53E-07	1
**Gene name**	SLO1	motifs												P	L	P	L	S	P	L	S	S	P	L	S	P	L2	S2				
**AGI number**	At2g22410	6												N	S	N	P	N	N	I	N	T	N	I	T	T	I	S				
**Type**	E	1'												D	N	D	V	D	D	T	T	D	D	N	N	D	Q	D				
**Species**	At	A												0.09	0.34	0.09	0.04	0.15	0.09	0.08	0.00	0.09	0.09	0.08	0.64	0.04	0.00	0.09				
**Localization**	mt	C												0.25	0.14	0.25	0.06	0.23	0.25	0.31	0.78	0.07	0.25	0.31	0.06	0.00	0.47	0.07				
**ref.**	23	G												0.14	0.12	0.14	0.17	0.07	0.14	0.16	0.14	0.74	0.14	0.16	0.15	0.93	0.21	0.70				
		U												0.52	0.40	0.52	0.73	0.55	0.52	0.45	0.09	0.10	0.52	0.45	0.16	0.03	0.33	0.14				
nad4	449	Target seq	A	C	U	A	U	U	C	U	A	U	G	U	U	U	U	U	U	U	C	G	A	A	A	G	C	G				
		score												0.52	0.40	0.52	0.73	0.55	0.52	0.45	0.78	0.74	0.09	0.08	0.64	0.93	0.47	0.70	3.22E-05	1	1.73E-06	1
nad9	328	Target seq	C	G	G	U	A	G	U	A	A	G	U	C	U	A	U	U	U	C	C	A	U	C	A	G	C	C				
		score												0.25	0.40	0.09	0.73	0.55	0.52	0.31	0.78	0.09	0.52	0.31	0.64	0.93	0.47	0.07	6.39E-04	4	1.44E-04	3
**Gene name**	SLO2	motifs											L	S	S	P	L	S	P	L	S	S	P	L	S	P	L2	S2				
**AGI number**	At2g13600	6											A	N	N	N	A	S	N	A	N	T	N	A	N	N	I	T				
**Type**	E	1'											E	N	D	N	D	N	D	D	N	N	T	D	D	D	L	D				
**Species**	At	A											0.25	0.14	0.15	0.08	0.25	0.70	0.09	0.25	0.14	0.64	0.04	0.25	0.15	0.09	0.00	0.09				
**Localization**	mt	C											0.25	0.29	0.23	0.55	0.25	0.14	0.25	0.25	0.29	0.06	0.69	0.25	0.23	0.25	0.47	0.07				
**ref.**	24	G											0.25	0.41	0.07	0.08	0.25	0.04	0.14	0.25	0.41	0.15	0.00	0.25	0.07	0.14	0.21	0.70				
		U											0.25	0.16	0.55	0.28	0.25	0.13	0.52	0.25	0.16	0.16	0.27	0.25	0.55	0.52	0.33	0.14				
nad4L	110	Target seq	U	A	U	U	C	U	U	A	U	U	A	U	G	U	U	A	A	U	G	U	C	A	A	U	U	G				
		score											0.25	0.16	0.07	0.28	0.25	0.70	0.09	0.25	0.41	0.16	0.69	0.25	0.15	0.52	0.33	0.70	5.28E-02	121	1.88E-02	48
nad7	739	Target seq	C	A	C	C	U	U	A	C	G	A	U	G	U	U	U	A	U	G	A	C	C	A	A	U	C	G				
		score											0.25	0.41	0.55	0.28	0.25	0.70	0.52	0.25	0.14	0.06	0.69	0.25	0.15	0.52	0.47	0.70	5.88E-03	11	1.05E-03	4
orfX	144	Target seq	G	G	U	U	U	G	A	C	A	U	G	G	U	U	U	A	C	G	U	G	U	U	A	C	U	G				
		score											0.25	0.41	0.55	0.28	0.25	0.70	0.25	0.25	0.16	0.15	0.27	0.25	0.15	0.25	0.33	0.70	1.36E-02	29	3.75E-03	10
orfX	145	Target seq	G	U	U	U	G	A	C	A	U	G	G	U	U	U	A	C	G	U	G	U	U	A	C	U	G	G				
		score											0.25	0.16	0.55	0.28	0.25	0.14	0.14	0.25	0.41	0.16	0.27	0.25	0.23	0.52	0.21	0.70	2.91E-02	68	1.25E-02	32
**Gene name**	MEF32	motifs																	S	P	L	S	P	L	S	P	L2	S2				
**AGI number**	AT4g14170	6																	S	N	P	S	T	V	N	S	L	A				
**Type**	E	1'																	N	D	S	D	D	G	D	N	E	D				
**Species**	At	A																	0.70	0.09	0.04	0.06	0.04	0.25	0.15	0.73	0.20	0.09				
**Localization**	mt	C																	0.14	0.25	0.06	0.00	0.00	0.25	0.23	0.13	0.38	0.07				
**ref.**	this paper	G																	0.04	0.14	0.17	0.85	0.93	0.25	0.07	0.00	0.06	0.70				
		U																	0.13	0.52	0.73	0.10	0.03	0.25	0.55	0.14	0.36	0.14				
ccmB	569	Target seq	U	C	A	U	G	U	U	C	U	U	U	U	A	U	U	G	A	U	U	G	G	U	U	A	U	U				
		score																	0.70	0.52	0.73	0.85	0.93	0.25	0.55	0.73	0.36	0.14	5.88E-05	2	3.35E-05	2
cox2	27	Target seq	A	A	A	A	U	G	A	U	U	G	U	U	C	U	A	A	A	A	U	G	G	U	U	A	U	U				
		score																	0.70	0.09	0.73	0.85	0.93	0.25	0.55	0.73	0.36	0.14	8.03E-04	4	2.92E-04	3
nad1	571	Target seq	U	U	C	C	U	C	U	G	U	U	C	C	C	U	G	U	A	U	U	G	G	U	U	A	U	G				
		score																	0.70	0.52	0.73	0.85	0.93	0.25	0.55	0.73	0.36	0.70	4.38E-05	1	1.59E-06	1
**Gene name**	OTP71	motifs														S	P	L	S	P	L	S	P	L	S	P	L2	S2				
**AGI number**	At1g64310	6														T	N	A	S	N	V	C	S	A	S	N	S	V				
**Type**	E	1'														S	D	D	D	N	H	D	D	D	N	D	Q	D				
**Species**	At	A														0.45	0.09	0.25	0.06	0.08	0.25	0.08	0.11	0.25	0.70	0.09	0.25	0.09				
**Localization**	mt	C														0.19	0.25	0.25	0.00	0.55	0.25	0.51	0.34	0.25	0.14	0.25	0.25	0.07				
**ref.**	25	G														0.17	0.14	0.25	0.85	0.08	0.25	0.34	0.45	0.25	0.04	0.14	0.25	0.70				
		U														0.18	0.52	0.25	0.10	0.28	0.25	0.07	0.10	0.25	0.13	0.52	0.25	0.14				
ccmFN2	176	Target seq	U	U	G	G	U	G	G	U	U	U	U	U	A	A	C	C	G	U	A	G	G	C	A	U	C	U				
		score														0.45	0.25	0.25	0.85	0.28	0.25	0.34	0.45	0.25	0.70	0.52	0.25	0.14	1.60E-04	2	4.39E-04	5

		PPR position	26	25	24	23	22	21	20	19	18	17	16	15	14	13	12	11	10	9	8	7	6	5	4	3	2	1	P-value	Rank without L, L2 and S2	P-value	Rank with L, L2 and S2
**Gene name**	OTP72	motifs										L	S	P	L	S	P	L	S	P	L	S	P	L	S	P	L2	S2				
**AGI number**	At3g13880	6										K	N	N	A	N	N	G	T	N	S	S	T	S	T	S	L	T				
**Type**	E	1'										C	N	D	Q	D	T	D	N	S	D	D	E	F	D	N	N	H				
**Species**	At	A										0.25	0.14	0.09	0.25	0.15	0.04	0.25	0.64	0.05	0.25	0.06	0.35	0.25	0.09	0.73	0.20	0.25				
**Localization**	mt	C										0.25	0.29	0.25	0.25	0.23	0.69	0.25	0.06	0.87	0.25	0.00	0.21	0.25	0.07	0.13	0.38	0.25				
**ref.**	25	G										0.25	0.41	0.14	0.25	0.07	0.00	0.25	0.15	0.00	0.25	0.85	0.22	0.25	0.74	0.00	0.06	0.25				
		U										0.25	0.16	0.52	0.25	0.55	0.27	0.25	0.16	0.08	0.25	0.10	0.22	0.25	0.10	0.14	0.36	0.25				
rpl16	440	Target seq	G	A	U	U	G	C	U	C	G	U	G	U	G	U	C	C	A	C	G	G	G	A	C	A	A	A				
		score										0.25	0.41	0.52	0.25	0.55	0.69	0.25	0.64	0.87	0.25	0.85	0.22	0.25	0.07	0.73	0.20	0.25	3.58E-05	2	5.00E-05	2
**Gene name**	MEF25	motifs														S	P	L	S	P	L	S	P	L	S	P	L2	S2				
**AGI number**	At3g25060	6														R	N	T	S	T	L	T	G	V	T	N	A	V				
**Type**	E	1'														G	D	D	D	D	N	T	D	D	D	D	S	A				
**Species**	At	A														0.25	0.09	0.40	0.06	0.04	0.20	0.45	0.25	0.25	0.09	0.09	0.25	0.25				
**Localization**	mt	C														0.25	0.25	0.19	0.00	0.00	0.32	0.19	0.25	0.25	0.07	0.25	0.25	0.25				
**ref.**	26	G														0.25	0.14	0.28	0.85	0.93	0.00	0.17	0.25	0.25	0.74	0.14	0.25	0.25				
		U														0.25	0.52	0.14	0.10	0.03	0.48	0.18	0.25	0.25	0.10	0.52	0.25	0.25				
nad1	308	Target seq	U	U	U	U	G	A	U	U	A	U	G	G	U	A	U	G	G	U	A	U	U	G	U	C	A	G				
		score														0.25	0.52	0.28	0.85	0.03	0.20	0.18	0.25	0.25	0.10	0.25	0.25	0.25	4.62E-02	76	4.11E-02	73
nad1	308 (-5)	Target seq	C	U	U	U	U	G	A	U	U	A	U	G	G	U	A	U	G	G	U	A	U	U	G	U	C	A				
		score														0.25	0.09	0.14	0.85	0.93	0.48	0.45	0.25	0.25	0.74	0.52	0.25	0.25	3.02E-01	4	1.20E-03	3
**Gene name**	PpPPR_56	motifs													L	S	P	L	S	P	L	S	P	L	S	P	L2	S2				
**AGI number**	Phypa_1694	6													V	N	N	M	T	T	V	T	N	L	T	N	V	N				
**Type**	DYW	1'													N	S	N	D	D	N	D	D	D	D	N	D	D	D				
**Species**	Pp	A													0.34	0.13	0.08	0.11	0.09	0.73	0.25	0.09	0.09	0.20	0.64	0.09	0.27	0.09				
**Localization**	mt	C													0.14	0.50	0.55	0.17	0.07	0.05	0.25	0.07	0.25	0.32	0.06	0.25	0.21	0.07				
**ref.**	28	G													0.12	0.09	0.08	0.22	0.74	0.22	0.25	0.74	0.14	0.00	0.15	0.14	0.09	0.70				
		U													0.40	0.28	0.28	0.51	0.10	0.00	0.25	0.10	0.52	0.48	0.16	0.52	0.42	0.14				
nad3	eU230S	Target seq	U	A	U	U	A	U	A	U	U	U	G	A	U	U	U	G	G	A	A	G	U	C	A	C	C	U				
		score													0.40	0.28	0.28	0.22	0.74	0.73	0.25	0.74	0.52	0.32	0.64	0.25	0.21	0.14	1.81E-04	1	2.69E-04	1
nad4	eU272S	Target seq	U	U	U	U	U	A	U	A	U	A	G	G	U	A	U	A	G	A	C	G	G	U	A	U	C	U				
		score													0.40	0.13	0.28	0.11	0.74	0.73	0.25	0.74	0.14	0.48	0.64	0.52	0.21	0.14	1.14E-03	2	1.72E-03	2
**Gene name**	PpPPR_71	motifs									P	L	S	P	L	S	P	L	S	P	L	S	P	L	S	P	L2	S2				
**AGI number**	Phypa_1813	6									P	V	N	N	L	T	N	L	T	T	M	T	S	I	N	N	V	G				
**Type**	DYW	1'									D	N	T	S	D	D	N	D	D	D	D	D	D	H	D	N	T	T				
**Species**	Pp	A									0.25	0.34	0.00	0.05	0.20	0.09	0.08	0.20	0.09	0.04	0.11	0.09	0.11	0.08	0.15	0.08	0.27	0.23				
**Localization**	mt	C									0.25	0.14	0.78	0.87	0.32	0.07	0.55	0.32	0.07	0.00	0.17	0.07	0.34	0.31	0.23	0.55	0.21	0.18				
**ref.**	29	G									0.25	0.12	0.14	0.00	0.00	0.74	0.08	0.00	0.74	0.93	0.22	0.74	0.45	0.16	0.07	0.08	0.09	0.00				
		U									0.25	0.40	0.09	0.08	0.48	0.10	0.28	0.48	0.10	0.03	0.51	0.10	0.10	0.45	0.55	0.28	0.42	0.60				
ccmFC	eU122SFue	Target seq	U	A	G	A	G	A	U	G	U	U	C	C	C	A	C	A	G	G	U	G	C	U	C	C	U	U				
		score									0.25	0.40	0.78	0.87	0.32	0.09	0.55	0.20	0.74	0.93	0.51	0.74	0.34	0.45	0.23	0.55	0.42	0.60	4.11E-05	1	8.58E-07	1
ccmFC	eU122SF	Target seq	U	A	G	A	G	A	U	G	U	U	U	C	C	A	C	A	G	G	U	G	C	U	C	C	U	U				
		score									0.25	0.40	0.09	0.87	0.32	0.09	0.55	0.20	0.74	0.93	0.51	0.74	0.34	0.45	0.23	0.55	0.42	0.60	4.17E-04	1	1.45E-05	1
**Gene name**	PpPPR_77	motifs	L	S	P	L	S	P	L	S	P	L	S	P	L	S	P	L	S	P	L	S	P	L	S	P	L2	S2				
**AGI number**	Phypa_1918	6	V	L	N	I	N	N	I	T	N	L	N	N	L	N	N	A	N	T	S	N	N	V	A	N	T	G				
**Type**	DYW	1'	D	D	N	D	D	D	D	D	N	D	D	G	N	D	D	D	D	V	D	D	N	D	N	D	T	D				
**Species**	Pp	A	0.25	0.08	0.08	0.08	0.15	0.09	0.08	0.09	0.08	0.20	0.15	0.25	0.20	0.15	0.09	0.25	0.15	0.35	0.25	0.15	0.08	0.25	0.53	0.09	0.70	0.09				
**Localization**	mt	C	0.25	0.51	0.55	0.31	0.23	0.25	0.31	0.07	0.55	0.32	0.23	0.25	0.32	0.23	0.25	0.25	0.23	0.21	0.25	0.23	0.55	0.25	0.00	0.25	0.16	0.07				
**ref.**	27	G	0.25	0.34	0.08	0.16	0.07	0.14	0.16	0.74	0.08	0.00	0.07	0.25	0.00	0.07	0.14	0.25	0.07	0.22	0.25	0.07	0.08	0.25	0.00	0.14	0.14	0.70				
		U	0.25	0.07	0.28	0.45	0.55	0.52	0.45	0.10	0.28	0.48	0.55	0.25	0.48	0.55	0.52	0.25	0.55	0.22	0.25	0.55	0.28	0.25	0.47	0.52	0.00	0.14				
cox2	eU370RW	Target seq	C	A	A	U	U	A	C	U	A	U	C	A	A	A	G	C	U	A	U	U	G	G	A	C	A	U				
		score	0.25	0.08	0.08	0.45	0.55	0.09	0.31	0.10	0.08	0.48	0.23	0.25	0.20	0.15	0.14	0.25	0.55	0.35	0.25	0.55	0.08	0.25	0.53	0.25	0.70	0.14	1.22E-04	3	2.58E-02	2
cox3	eU733RW	Target seq	A	C	U	U	U	G	G	C	U	U	U	G	A	A	G	C	A	G	C	U	G	C	U	U	G	G				
		score	0.25	0.51	0.28	0.45	0.55	0.14	0.16	0.07	0.28	0.48	0.55	0.25	0.20	0.15	0.14	0.25	0.15	0.22	0.25	0.55	0.08	0.25	0.47	0.52	0.14	0.70	1.33E-02	1	3.22E-03	1
**Gene name**	PpPPR_78	motifs						P	L	S	P	L	S	P	L	S	P	L	S	P	L	S	P	L	S	P	L2	S2				
**AGI number**	Phypa_1926	6						F	M	N	N	M	N	T	I	T	N	V	N	T	T	N	N	I	N	N	V	G				
**Type**	DYW	1'						N	D	T	D	D	S	N	D	D	N	D	D	N	D	D	D	D	N	D	T	N				
**Species**	Pp	A						0.17	0.11	0.00	0.09	0.11	0.13	0.73	0.08	0.09	0.08	0.25	0.15	0.73	0.40	0.15	0.09	0.08	0.14	0.09	0.27	0.91				
**Localization**	mt	C						0.44	0.17	0.78	0.25	0.17	0.50	0.05	0.31	0.07	0.55	0.25	0.23	0.05	0.19	0.23	0.25	0.31	0.29	0.25	0.21	0.00				
**ref.**	30	G						0.00	0.22	0.14	0.14	0.22	0.09	0.22	0.16	0.74	0.08	0.25	0.07	0.22	0.28	0.07	0.14	0.16	0.41	0.14	0.09	0.00				
		U						0.39	0.51	0.09	0.52	0.51	0.28	0.00	0.45	0.10	0.28	0.25	0.55	0.00	0.14	0.55	0.52	0.45	0.16	0.52	0.42	0.09				
cox1	eU755SL	Target seq	U	C	C	C	G	A	U	C	C	U	G	A	G	G	U	C	U	A	U	A	U	U	C	U	A	A				
		score						0.17	0.51	0.78	0.25	0.51	0.09	0.73	0.16	0.74	0.28	0.25	0.55	0.73	0.14	0.15	0.52	0.45	0.29	0.52	0.27	0.91	5.30E-04	2	4.71E-05	1
rps14	eU137SL	Target seq	U	C	A	A	A	U	A	C	G	U	U	A	U	G	A	A	U	A	U	U	U	U	U	U	A	A				
		score						0.39	0.11	0.78	0.14	0.51	0.28	0.73	0.45	0.74	0.08	0.25	0.55	0.73	0.14	0.55	0.52	0.45	0.16	0.52	0.27	0.91	3.44E-04	1	5.46E-05	2
**Gene name**	PpPPR_79	motifs										L	S	P	L	S	P	L	S	P	L	S	P	L	S	P	L2	S2				
**AGI number**	Phypa_1927	6										V	N	N	V	N	T	M	T	N	T	N	T	M	N	N	V	A				
**Type**	DYW	1'										N	S	D	D	D	S	D	D	D	D	D	N	D	D	N	T	S				
**Species**	Pp	A										0.34	0.13	0.09	0.25	0.15	0.35	0.11	0.09	0.09	0.40	0.15	0.73	0.11	0.15	0.08	0.27	0.25				
**Localization**	mt	C										0.14	0.50	0.25	0.25	0.23	0.21	0.17	0.07	0.25	0.19	0.23	0.05	0.17	0.23	0.55	0.21	0.25				
**ref.**	30	G										0.12	0.09	0.14	0.25	0.07	0.22	0.22	0.74	0.14	0.28	0.07	0.22	0.22	0.07	0.08	0.09	0.25				
		U										0.40	0.28	0.52	0.25	0.55	0.22	0.51	0.10	0.52	0.14	0.55	0.00	0.51	0.55	0.28	0.42	0.25				
nad5	eU598RC	Target seq	A	A	A	C	A	G	U	A	G	A	C	U	U	U	U	C	G	A	C	U	A	U	U	U	U	U				
		score										0.34	0.50	0.52	0.25	0.55	0.22	0.17	0.74	0.09	0.19	0.55	0.73	0.51	0.55	0.28	0.42	0.25	2.60E-04	1	1.00E-04	1
**Gene name**	PpPPR_91	motifs									L	S	P	L	S	P	L	S	P	L	S	S	P	L	S	P	L2	S2				
**AGI number**	Phypa_2112	6									G	N	S	L	T	T	V	N	S	M	T	T	N	I	T	N	T	G				
**Type**	DYW	1'									D	D	N	D	N	N	D	D	N	D	N	N	D	D	D	N	R	D				
**Species**	Pp	A									0.25	0.15	0.73	0.20	0.64	0.73	0.25	0.15	0.73	0.11	0.64	0.64	0.09	0.08	0.09	0.08	0.70	0.09				
**Localization**	mt	C									0.25	0.23	0.13	0.32	0.06	0.05	0.25	0.23	0.13	0.17	0.06	0.06	0.25	0.31	0.07	0.55	0.16	0.07				
**ref.**	27	G									0.25	0.07	0.00	0.00	0.15	0.22	0.25	0.07	0.00	0.22	0.15	0.15	0.14	0.16	0.74	0.08	0.14	0.70				
		U									0.25	0.55	0.14	0.48	0.16	0.00	0.25	0.55	0.14	0.51	0.16	0.16	0.52	0.45	0.10	0.28	0.00	0.14				
nad5	eU730RW	Target seq	G	A	A	A	A	U	C	U	G	C	A	C	A	A	A	U	A	G	G	A	U	U	G	C	A	U				
		score									0.25	0.23	0.73	0.32	0.64	0.73	0.25	0.55	0.73	0.22	0.15	0.64	0.52	0.45	0.74	0.55	0.70	0.14	6.18E-06	1	2.73E-06	1

		PPR position	26	25	24	23	22	21	20	19	18	17	16	15	14	13	12	11	10	9	8	7	6	5	4	3	2	1	P-value	Rank without L, L2 and S2	P-value	Rank with L, L2 and S2
**Gene name**	OGR1	motifs																			P	P	P	L	S	P	L2	S2				
**AGI number**	OS12g17080	6																			S	T	G	V	N	N	L	G				
**Type**	DYW	1'																			D	N	N	N	T	D	N	D				
**Species**	Os	A																			0.11	0.73	0.17	0.34	0	0.09	0.2	0.09				
**Localization**	mt	C																			0.34	0.05	0.44	0.14	0.78	0.25	0.38	0.07				
**ref.**	31	G																			0.45	0.22	0	0.12	0.14	0.14	0.06	0.7				
		U																			0.1	0	0.39	0.4	0.09	0.52	0.36	0.14				
cox2	167	Target seq	C	U	U	C	C	U	U	A	U	U	C	U	G	A	U	U	U	U	G	G	U	U	U	U	C	G				
		score																			0.45	0.22	0.39	0.40	0.09	0.52	0.38	0.70	2.44E-02	48	3.86E-03	12
cox3	572	Target seq	A	G	U	A	G	C	A	A	C	C	G	U	U	U	U	A	C	U	G	G	C	U	C	U	A	G				
		score																			0.45	0.22	0.44	0.40	0.78	0.52	0.20	0.70	2.44E-03	5	4.70E-04	1
nad4	458	Target seq	A	A	A	G	C	U	U	U	C	U	G	U	C	G	A	A	C	U	G	G	C	U	U	C	U	A				
		score																			0.45	0.73	0.39	0.14	0.14	0.25	0.38	0.70	7.05E-02	70	2.67E-02	71
ccmC	1457	Target seq	C	G	A	U	C	U	U	A	C	U	C	A	U	C	A	A	A	U	G	G	C	A	C	U	C	A				
		score																			0.45	0.22	0.44	0.40	0.09	0.25	0.36	0.09	1.88E-03	4	2.46E-03	5
ccmC	401	Target seq	A	A	U	U	U	G	U	G	A	A	U	U	U	C	U	A	A	U	G	A	U	C	G	C	C	G				
		score																			0.45	0.22	0.44	0.40	0.78	0.25	0.36	0.09	8.02E-03	9	3.01E-03	6
nad2	416	Target seq	A	A	U	G	A	U	C	G	C	C	G	U	G	U	U	C	U	G	C	A	U	G	C	U	G	G				
		score																			0.45	0.22	0.44	0.34	0.78	0.52	0.38	0.09	5.34E-04	2	3.20E-03	9
nad4	433	Target seq	G	C	A	U	G	C	U	G	G	A	U	C	U	U	C	U	A	C	U	A	U	U	C	U	A	U				
		score																			0.34	0.73	0.39	0.12	0.78	0.52	0.06	0.70	8.02E-03	10	7.97E-03	20
nad4	458ue	Target seq	A	A	A	G	C	U	U	U	C	U	G	U	C	G	A	A	C	U	G	G	C	U	C	C	U	A				
		score																			0.10	0.73	0.39	0.40	0.78	0.52	0.20	0.14	5.77E-03	6	5.20E-03	15

**Gene name**	MPR25	motifs								S	P	L	S	P	L	S	P	L	S	P	L	S	P	L	S	P	L2	S2				
**AGI number**	Os04g51350	6								A	N	S	N	N	V	S	N	L	N	N	T	S	N	A	T	T	L	C				
**Type**	E	1'								P	D	T	N	T	D	N	D	P	D	D	G	D	T	N	S	D	T	N				
**Species**	Os	A								0.25	0.09	0.25	0.14	0.04	0.25	0.70	0.09	0.20	0.15	0.09	0.40	0.06	0.04	0.34	0.45	0.04	0.20	0.91				
**Localization**	mt	C								0.25	0.25	0.25	0.29	0.69	0.25	0.14	0.25	0.32	0.23	0.25	0.19	0.00	0.69	0.14	0.19	0.00	0.38	0.00				
**ref.**	32	G								0.25	0.14	0.25	0.41	0.00	0.25	0.04	0.14	0.00	0.07	0.14	0.28	0.85	0.00	0.12	0.17	0.93	0.06	0.00				
		U								0.25	0.52	0.25	0.16	0.27	0.25	0.13	0.52	0.48	0.55	0.52	0.14	0.10	0.27	0.40	0.18	0.03	0.36	0.09				
nad5	1580	Target seq	C	A	A	A	C	U	A	A	U	A	C	C	U	A	U	U	C	U	G	U	U	U	A	G	U	A				
		score								0.25	0.52	0.25	0.29	0.69	0.25	0.70	0.52	0.48	0.23	0.52	0.28	0.10	0.27	0.40	0.45	0.93	0.36	0.91	4.17E-04	3	1.96E-05	2

Figure S3
