# Supplementary figures and images for: Improved Computational Target Site Prediction for Pentatricopeptide Repeat RNA Editing Factors
Source: PLoS One. 2013 Jun 6;8(6):e65343. doi: 10.1371/journal.pone.0065343 (PMC3675099; doi:10.1371/journal.pone.0065343)

P

position 6 & position 1'

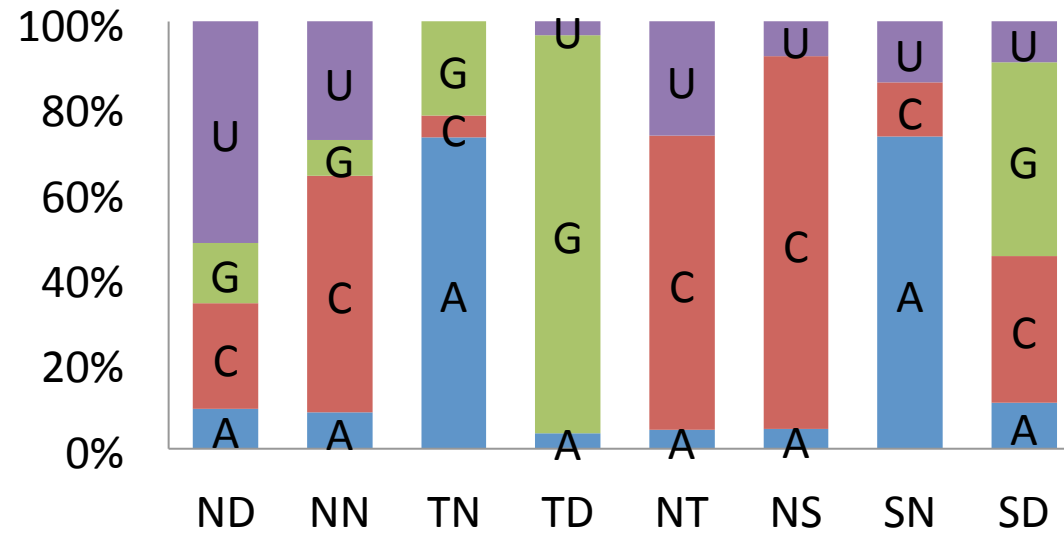

S

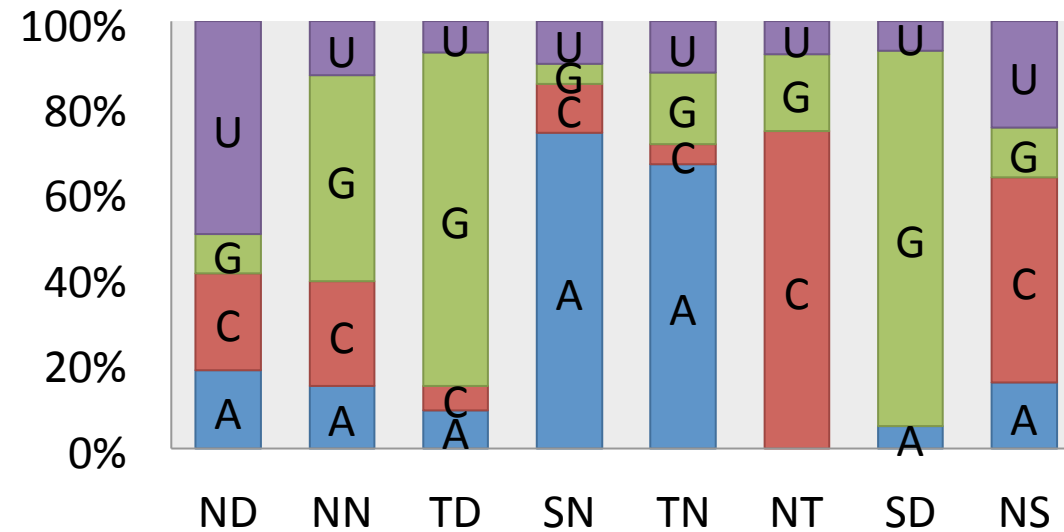

Figure S4

Supplement: Figure S4 — Correlation between amino acid combinations at positions 6 and 1′ in P and S motifs and nucleotide identities. The amino acid combinations are given in the order 6 and 1′. Displayed are the percentages of coincidences between a given amino acid combination and the nucleotide identity. Amino acid combinations are shown from left to right ordered by their number of occurrence. These data are compiled from data as shown in figure S2C. For the P-elements the combinations N6D1’, N6N1’, T6N1’, T6D1’, N6T1’, N6S1’, S6N1’ and S6D1’ show the strongest correlations. (PDF) [file pone.0065343.s004.pdf]

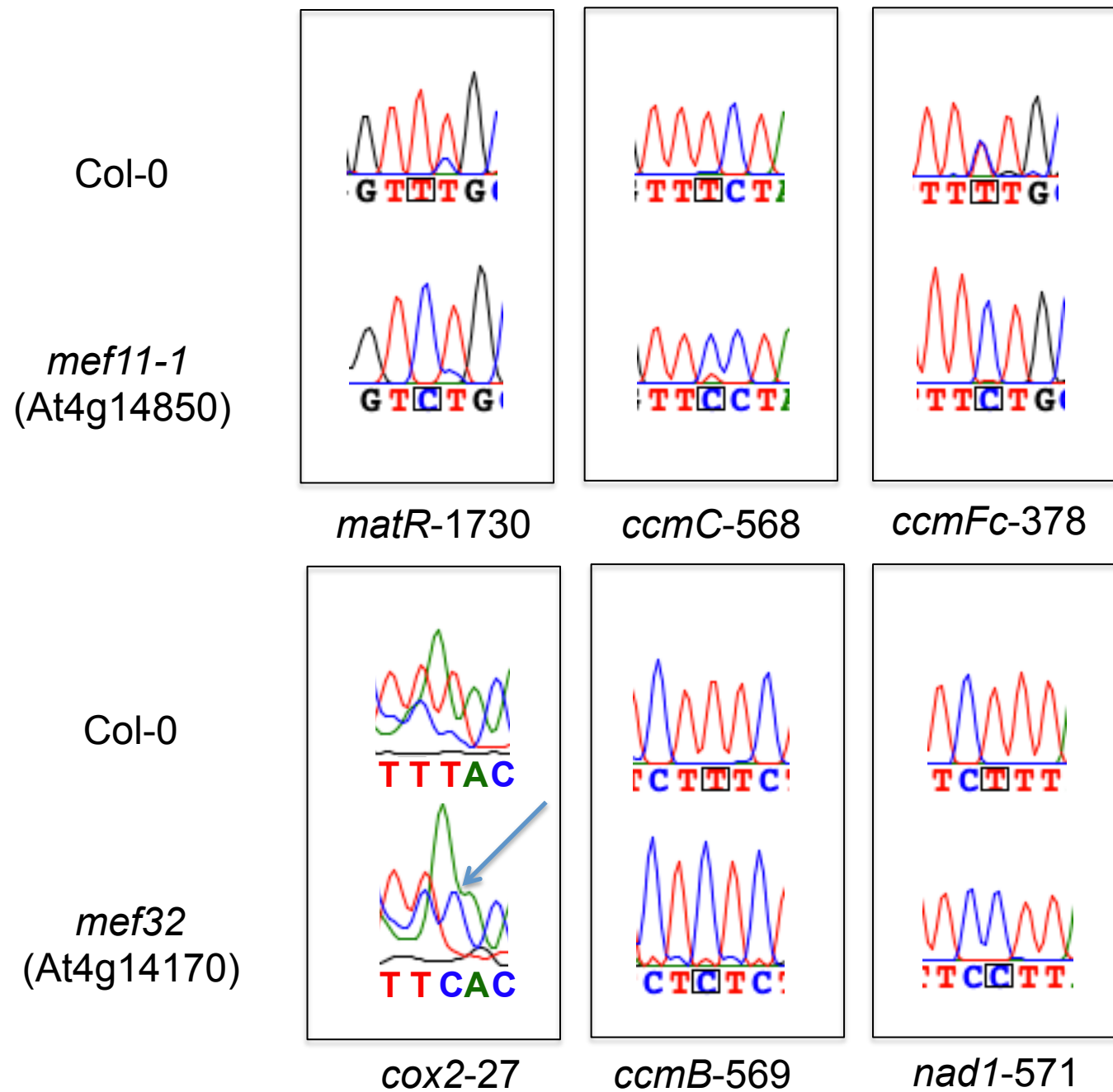

Figure S5

Supplement: Figure S5 — Target sites predicted for MEF11 and MEF32 are analysed in respective mutant plants. The top panels show a comparison of the cDNA sequences at the new target sites predicted for MEF11 between Col-0 wild type plants and the knock-out mutant mef11-1. While in the wild type plants the genomic encoded C is changed to T in the cDNA, the C remains unedited in mutant mef11-1. Site ccmFc-378 (ccb452-378) is a silent nucleotide exchange and is edited to only about 40% in Col-0 wild type plants. The lower panels show a comparison between the cDNA sequences at the target sites predicted for the previously unassigned PPR RNA editing factor MEF32 between Col-0 wild type plants and the knock-out mutant mef32. While in the wild type plants the genomic encoded C is changed to T in the cDNA, the C remains unedited in the mutant. The arrow points to the C peak not present in the wild type plants. (PDF) [file pone.0065343.s005.pdf]

A

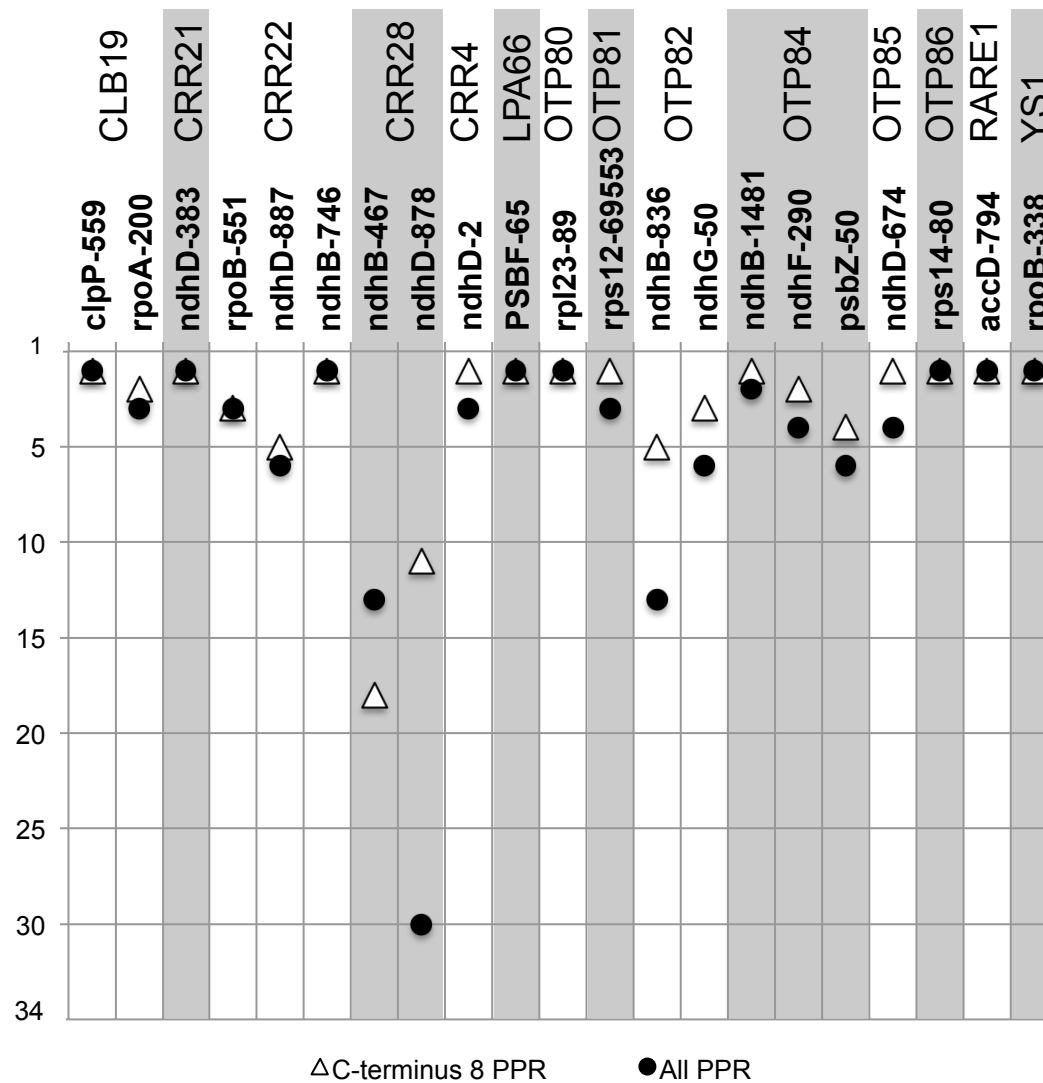

Figure S6A

B

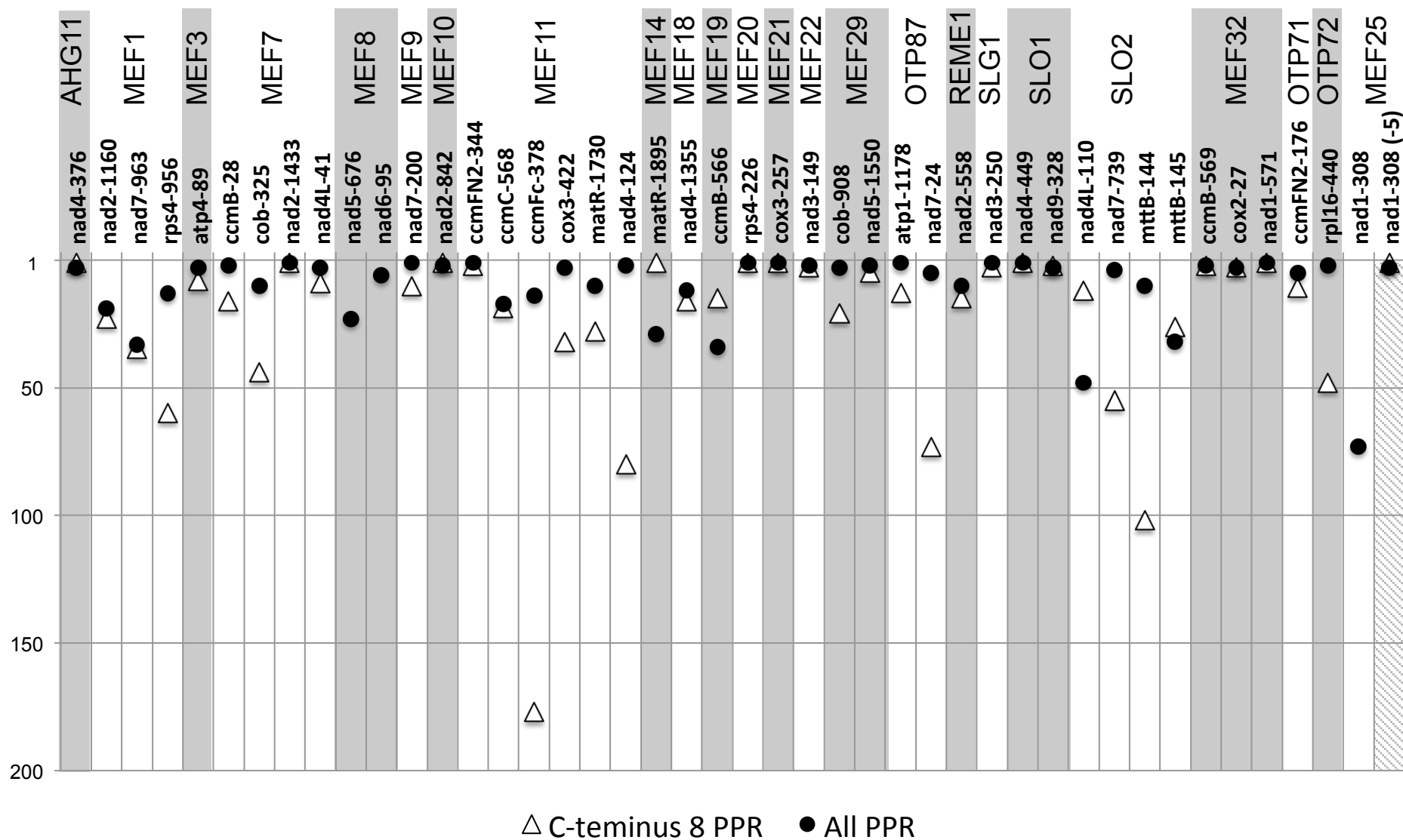

Figure S6B

Supplement: Figure S6 — The eight C-terminal PPR elements including the L, L2 and S2 repeats are often sufficient to predict RNA editing targets. (A) Prediction of the target sites for the known chloroplast editing factors of Arabidopsis identifies bona fide targets within the top ranks from only the eight PPR elements at the C-terminus of the respective protein. Actually the ranking within the 34 RNA editing sites in chloroplasts is often better with only these eight PPR elements than when all PPR elements are included. (B) Analogous comparative analysis of the predictions within 430 editing sites considered for mitochondrial PPR proteins usually shows less faithful ranking with only the eight PPR elements at the C-terminus of the respective protein. Only in a few instances such as MEF14 and some sites of SLO2 the predicted PPR-RNA interaction is increased in rank in comparison to the prediction from all PPR elements. (PDF) [file pone.0065343.s006.pdf]

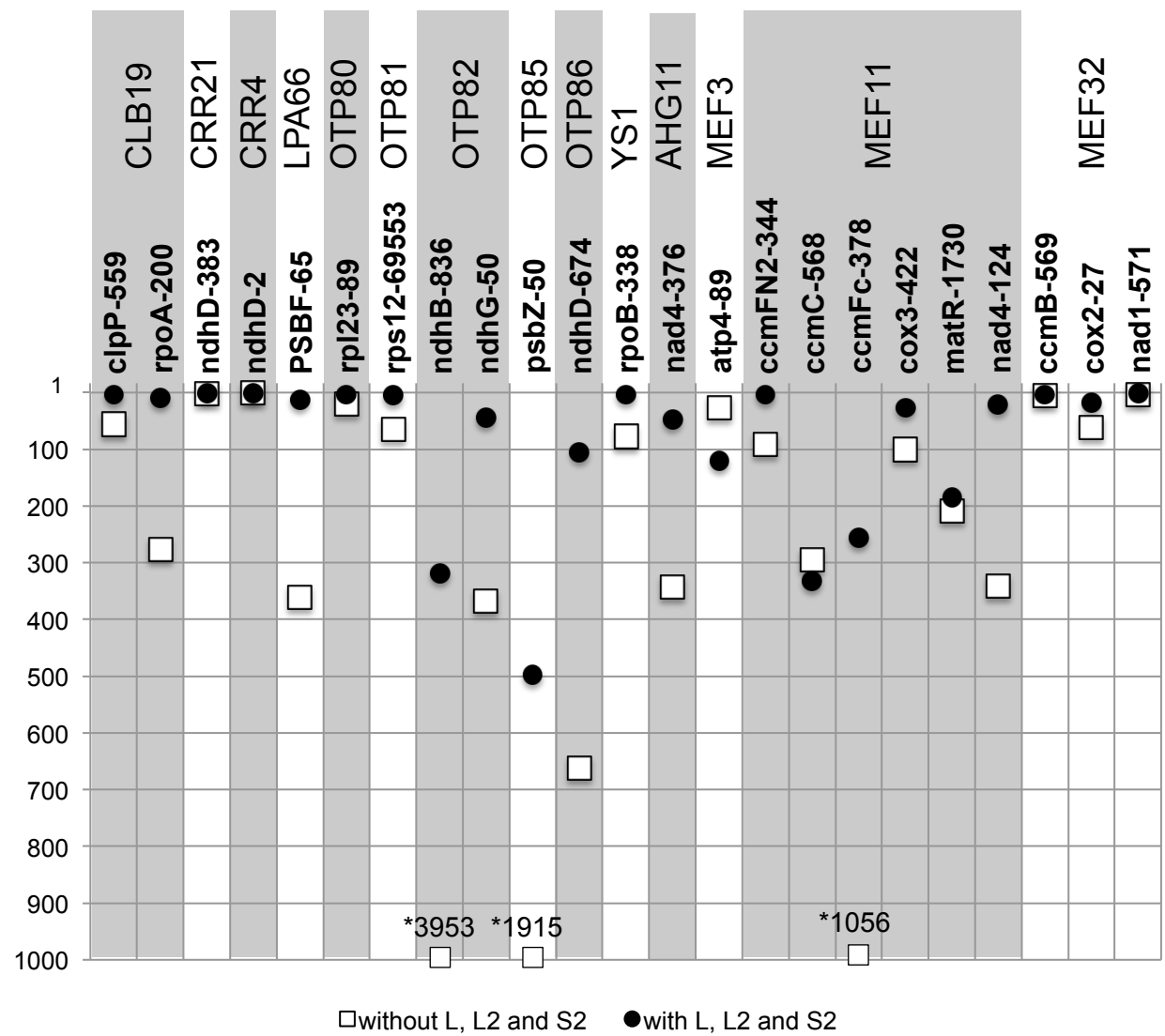

Figure S7

Supplement: Figure S7 — Inclusion of L, L2 and S2 repeats generally improves the prediction accuracy of RNA editing targets in the respective entire transcriptome. The bona fide RNA editing target sites will have to be identified in vivo by the PPR protein factor against the presence of all C nucleotides in the respective organelle. Screening the prediction accuracy within all C nucleotides with or without including the L, L2 and S2 repeats, their inclusion generally improves the ranking considerably. Shown here are data for selected RNA editing PPR proteins for plastids and mitochondria (MEFs) from Arabidopsis. Screening was done against all C nucleotides in all transcripts in plastids (17.886) and in the mitochondrial transcripts with known functions respectively, both as annotated in the Flagdb. Changes in the ranking predictions are seen for example with the novel mitochondrial PPR protein MEF32 for which rankings change from positions 5 to 3, from 61 to 18 and from 3 to 1 upon inclusion of the L, L2 and S2 repeats. For MEF11, the predicted PPR-RNA interactions change rank from positions 92 to 4, from 295 to 331, from 100 to 27, from 209 to 184 and from 342 to 21 when the L, L2 and S2 elements are included. Some target sites (asterisks) are not ranked in the top 1000. (PDF) [file pone.0065343.s007.pdf]
