# Supplementary material for: Improved Computational Target Site Prediction for Pentatricopeptide Repeat RNA Editing Factors
Source: PLoS One. 2013 Jun 6;8(6):e65343. doi: 10.1371/journal.pone.0065343 (PMC3675099; doi:10.1371/journal.pone.0065343)
Supplement: References S1 — (DOCX) [file pone.0065343.s008.docx]

**References S1**

1. Chateigner-Boutin A-L, Ramos-Vega M, Guevara-García A, Andrés C, de la Luz Gutiérrez-Nava Ma, et al. (2008) CLB19, a pentatricopeptide repeat protein required for editing of rpoA and clpP chloroplast transcripts. Plant J 56: 590-602.

2. Okuda K, Myouga F, Motohashi R, Shinozaki K, Shikanai T (2007) Conserved domain structure of pentatricopeptide repeat proteins involved in chloroplast RNA editing. Proc Natl Acad Sci USA 104: 8178-8183.

3. Okuda K, Chateigner-Boutin AL, Nakamura T, Delannoy E, Sugita M, et al. (2009) Pentatricopeptide repeat proteins with the DYW motif have distinct molecular functions in RNA editing and RNA cleavage in Arabidopsis chloroplasts. Plant Cell 21: 146-156.

4. Kotera E, Tasaka M, Shikanai T (2005) A pentatricopeptide repeat protein is essential for RNA editing in chloroplasts. Nature 433: 326-330.

5. Cai W, Ji D, Peng L, Guo J, Ma J, et al. (2009) LPA66 is required for editing psbF chloroplast transcripts in Arabidopsis. Plant Physiol 150: 1260-1271.

6. Hammani K, Okuda K, Tanz SK, Chateigner-Boutin AL, Shikanai T, et al. (2009) A study of new Arabidopsis chloroplast RNA editing mutants reveals general features of editing factors and their target sites. Plant Cell 21: 3686-3699.

7. Robbins JC, Heller WP, Hanson M (2009) A comparative genomics approach identifies a PPR-DYW protein that is essential for C-to-U editing of the Arabidopsis chloroplast accD transcript. RNA 15: 1142–1153.

8. Zhou W, Cheng Y, Yap A, Chateigner-Boutin A-L, Delannoy E, et al. (2009) The Arabidopsis gene YS1 encoding a DYW protein is required for editing of rpoB transcripts and the rapid development of chloroplasts during early growth. Plant J 58: 82-96.

9. Yagi Y, Hayashi S, Kobayashi K, Hirayama T, Nakamura T (2013) Elucidation of the RNA recognition code for pentatricopeptide repeat proteins involved in organelle RNA editing in plants. PLoS ONE 8: e57286.

10. Zehrmann A, Verbitskiy D, van der Merwe JA, Brennicke A, Takenaka M (2009) A DYW domain-containing pentatricopeptide repeat protein is required for RNA editing at multiple sites in mitochondria of Arabidopsis thaliana. Plant Cell 21: 558-567.

11. Verbitskiy D, Merwe JA, Zehrmann A, Hartel B, Takenaka M (2012) The E-class PPR protein MEF3 of Arabidopsis thaliana can also function in mitochondrial RNA editing with an additional DYW domain. Plant Cell Physiol 53: 358-367.

12. Zehrmann A, van der Merwe J, Verbitskiy D, Härtel B, Brennicke A, et al. (2012) The DYW-class PPR protein MEF7 is required for RNA editing at four sites in mitochondria of Arabidopsis thaliana. RNA Biol 9: 155-161.

13. Verbitskiy D, Zehrmann A, Härtel B, Brennicke A, Takenaka M (2012) Two related RNA editing proteins target the same sites in mitochondria of Arabidopsis thaliana. J Biol Chem 287: 38064-38072.

14. Takenaka M (2009) MEF9, an E-subclass pentatricopeptide repeat protein, is required for an RNA editing event in the nad7 transcript in mitochondria of Arabidopsis. Plant Physiol 152: 939-947.

15. Härtel B, Zehrmann A, Verbitskiy D, van der Merwe JA, Brennicke A, Takenaka M (2013) MEF10 is required for RNA editing at nad2-842 in mitochondria of Arabidopsis thaliana and interacts with MORF8. Plant Mol Biol 81: 337-346.

16. Verbitskiy D, Zehrmann A, van der Merwe JA, BrennickeA, Takenaka M (2010) The PPR protein encoded by the lovastatin insensitive 1 gene is involved in RNA editing at three sites in mitochondria of Arabidopsis thaliana. Plant J 61: 446-455.

17. Verbitskiy D, Härtel B, Zehrmann A, Brennicke A, Takenaka M (2011) The DYW-E-PPR protein MEF14 is required for RNA editing at site matR-1895 in mitochondria of Arabidopsis thaliana. FEBS Lett 585: 700-704.

18. Takenaka M, Verbitskiy D, Zehrmann A, Brennicke A (2010) Reverse genetic screening identifies five E-class PPR proteins involved in RNA editing in mitochondria of Arabidopsis thaliana. J Biol Chem 285: 27122-27129.

19. Sosso D, Mbelo S, Vernoud V, Gendrot G, Dedieu A, et al. (2012) PPR2263, a DYW-Subgroup Pentatricopeptide repeat protein, is required for mitochondrial nad5 and cob transcript editing, mitochondrion biogenesis, and maize growth. Plant Cell 24: 676-691.

20. Hammani K, Colas des Francs-Small C, Takenaka M, Tanz SK, Okuda K, et al. (2011) The pentatricopeptide repeat protein OTP87 is essential for RNA editing of nad7 and atp1 transcripts in Arabidopsis mitochondria. J Biol Chem 286: 21361-21371.

21. Bentolila S, Knight W, Hanson M (2010) Natural Variation in Arabidopsis Leads to the Identification of REME1, a Pentatricopeptide Repeat-DYW Protein Controlling the Editing of Mitochondrial Transcripts. Plant Physiol 154: 1966–1982.

22. Yuan H, Liu D (2012) Functional disruption of the pentatricopeptide protein SLG1 affects mitochondrial RNA editing, plant development, and responses to abiotic stresses in Arabidopsis. Plant J 70: 432-444.

23. Sung T-Y, Tseng C-C, Hsieh M-H (2010) The SLO1 PPR protein is required for RNA editing at multiple sites with similar upstream sequences in Arabidopsis mitochondria. Plant J 63: 499-511.

24. Zhu Q, Dugardeyn J, Zhang C, Takenaka M, Kühn K, et al. (2012) SLO2, a mitochondrial PPR protein affecting several RNA editing sites, is required for energy metabolism. Plant J 71: 836-849.

25. Chateigner-Boutin AL, [Colas des Francs-Small C](http://www.ncbi.nlm.nih.gov/pubmed?term=Colas%20des%20Francs-Small%20C%5BAuthor%5D&cauthor=true&cauthor_uid=23521509), [Fujii S](http://www.ncbi.nlm.nih.gov/pubmed?term=Fujii%20S%5BAuthor%5D&cauthor=true&cauthor_uid=23521509), [Okuda K](http://www.ncbi.nlm.nih.gov/pubmed?term=Okuda%20K%5BAuthor%5D&cauthor=true&cauthor_uid=23521509), [Tanz SK](http://www.ncbi.nlm.nih.gov/pubmed?term=Tanz%20SK%5BAuthor%5D&cauthor=true&cauthor_uid=23521509), [Small I](http://www.ncbi.nlm.nih.gov/pubmed?term=Small%20I%5BAuthor%5D&cauthor=true&cauthor_uid=23521509) (2013) The E domains of pentatricopeptide repeat proteins from different organelles are not functionally equivalent for RNA editing. Plant J doi: 10.1111/tpj.12180.

26. Arenas-MA, Takenaka M, Moreno S, Gómez I, Jordana X (2013) Contiguous RNA editing sites in the mitochondrial nad1 transcript of Arabidopsis thaliana are recognized by different proteins. FEBS Lett 587: 887-891.

27. Ohtani S, Ichinose M, Tasaki E, Aoki Y, Komura Y, et al. (2010) Targeted gene disruption identifies three PPR-DYW proteins involved in RNA editing for five editing sites of the moss mitochondrial transcripts. Plant Cell Physiol 51: 1942-1949.

28. Rüdinger M, Szovenyi P, Rensing SA, Knoop V (2011) Assigning DYW-type PPR proteins to RNA editing sites in the funariid mosses Physcomitrella patens and Funaria hygrometrica. Plant J 67: 370-380.

29. Tasaki E, Hattori M, Sugita M (2010) The moss pentatricopeptide repeat protein with a DYW domain is responsible for RNA editing of mitochondrial ccmFc transcript. Plant J 62: 560-570.

30. Uchida M, Ohtani S, Ichinose M, Sugita C, Sugita M (2011) The PPR-DYW proteins are required for RNA editing of rps14, cox1 and nad5 transcripts in Physcomitrella patens mitochondria. FEBS Lett 585: 2367-2371.

31. Kim S-R, Yang J-I, Moon S, Ryu C-H, An K, et al. (2009) Rice OGR1 encodes a pentatricopeptide repeat-DYW protein and is essential for RNA editing in mitochondria. Plant J 59: 738–749.

32. Toda T, Fujii S, Noguchi K, Kazama T, Toriyama K. (2012), Rice *MPR25* encodes a pentatricopeptide repeat protein and is essential for RNA editing of *nad5* transcripts in mitochondria. Plant J 72: 450–460.
