# Supplementary material for: Improved Computational Target Site Prediction for Pentatricopeptide Repeat RNA Editing Factors
Source: PLoS One. 2013 Jun 6;8(6):e65343. doi: 10.1371/journal.pone.0065343 (PMC3675099; doi:10.1371/journal.pone.0065343)
Supplement: Figure S1 — Evaluation of coincidences between amino acids within PPR elements and corresponding nucleotides. Part of the entire set of aligments is shown for the PPR editing protein MEF1. Each amino acid in each motif is analysed for co-occurences between amino acid and corresponding nucleotide identities. Amino acids at positions 1′ and 6 show the strongest correlations as depicted in figure 1. For the length of motif S2 convention assigns 36 nucleotides which positions amino acid 1′ into S2 (indicated by an arrow and shading), different from all other elements where amino acid 1′ is usually assigned to the first amino acid position of the C-terminally adjacent element. (PDF) [file pone.0065343.s001.pdf]

C

N

				position	1'	1	2	3	4	5	6	7	8	9	10	11	12	13	14	15	16	17	18	19	20	21	22	23	24	25	26	27	28	29	30	31	32	33	34	35	36				
At no	Gene name	motifs	PPR																																										
At5g52630	MEF1	S2	1		T	T	D	K	H	Y	A	S	L	V	D	M	L	G	R	A	G	R	L	Q	E	A	L	E	V	I	T	N	M	P	I	D	P	T	E	S		A	U	U	-4
At5g52630	MEF1	L2	2		T	N	F	I	T	F	L	N	V	L	N	A	C	S	H	A	G	L	V	D	E	G	R	Y	Y	F	D	Q	M	K	E	S	R	I	E	P		U	U	U	-5
At5g52630	MEF1	P	3		N	N	L	G	I	W	N	A	M	L	K	A	Y	A	Q	H	S	H	T	Q	K	V	I	E	L	F	K	R	M	K	L	S	G	M	K	P		C	U	A	-6
At5g52630	MEF1	S	4		N	S	S	F	V	G	S	S	L	V	S	L	Y	S	K	C	G	V	P	E	G	A	Y	Q	V	F	N	E	V	P	V	K						C	G	C	-7
At5g52630	MEF1	L	5		S	N	D	Y	S	F	S	S	V	I	S	V	C	A	N	S	T	L	L	E	L	G	R	Q	I	H	G	L	S	I	K	S	S	F	D	S		U	G	U	-8
At5g52630	MEF1	P	6		N	N	V	V	T	W	S	G	M	M	Y	G	Y	A	Q	M	G	E	N	E	E	A	L	W	L	F	K	E	A	L	F	E	N	L	A	V		A	A	C	-9
At5g52630	MEF1	S	7		N	D	V	F	V	G	S	S	L	V	D	M	Y	A	K	C	G	E	I	V	Y	A	R	K	M	F	D	E	M	P								A	A	A	-10
At5g52630	MEF1	L	8		D	D	D	H	V	L	P	S	A	T	K	S	C	A	I	L	S	R	C	D	I	G	R	S	V	H	C	L	S	M	K	T	G	Y	D	A		G	G	U	-11
At5g52630	MEF1	P	9		D	S	S	T	T	W	S	S	I	I	S	C	F	A	Q	N	E	L	P	W	M	S	L	E	F	L	K	K	M	M	A	G	N	L	R	P		C	A	C	-12
At5g52630	MEF1	S	10		S	I	P	L	V	A	N	N	L	I	N	F	Y	S	K	S	Q	L	P	F	D	S	R	R	A	F	E	D	S	P	Q	K						A	C	C	-13
At5g52630	MEF1	L	11		I	N	Y	N	Q	I	C	D	L	L	L	S	S	A	R	T	R	S	T	I	K	G	L	Q	L	H	G	Y	V	V	K	S	G	L	S	L		A	A	A	-14

mt genes	sites		-30										-20						-14	-13	-12	-11	-10	-9	-8	-7	-6	-5	-4				0					+5
nad2	1160		U	G	G	G	C	G	C	U	C	U	A	G	C	C	A	A	A	A	C	G	A	A	U	C	C	U	A	U	U	U	**C**	G	G	C	U	A
nad7	963		C	C	A	U	U	U	C	G	A	A	C	U	U	U	A	U	A	C	A	G	A	A	G	G	U	U	U	U	U	C	**C**	G	U	A	C	C
rps4	956		U	C	A	A	A	A	G	G	A	U	C	G	A	A	C	U	A	C	C	U	A	C	U	C	A	U	U	A	U	U	**C**	G	G	A	G	G

Figure S1
